# Impact of Standardised Packaging of Tobacco Products Regulations on cigarette consumption and youth smoking in England: interrupted time-series analysis

**DOI:** 10.1136/tc-2023-058560

**Published:** 2024-06-08

**Authors:** Vera Helen Buss, Loren Kock, Emma Beard, Lion Shahab, Jamie Brown, Sarah Jackson

**Affiliations:** 1 Department of Behavioural Science and Health, University College London, London, UK; 2 SPECTRUM Research Consortium, Edinburgh, UK; 3 Department of Psychiatry, University of Vermont, Burlington, Vermont, USA; 4 Department of Epidemiology and Public Health, University College London, London, UK

**Keywords:** Hand-rolled/RYO tobacco, Packaging and Labelling, Public policy, Harm Reduction

## Abstract

**Background:**

In the UK in May 2016, standardised packaging of tobacco products was implemented, including minimum pack sizes of 20 sticks or 30 g loose tobacco. The change was intended to reduce uptake by increasing upfront costs to young people, but there was concern it may unintentionally increase consumption among people smoking. This study aimed to assess whether the introduction of the policy was associated with changes in (1) mean daily factory-made (FM)/roll-your-own (RYO) cigarettes consumption among people smoking predominantly (a) FM and (b) RYO cigarettes; and (2) current smoking prevalence among 16–24-year-olds.

**Methods:**

Data (N=257 929) were from a representative monthly cross-sectional survey of adults (≥16 years) in England, collected between November 2007 and January 2020. Outcome measures were mean daily (FM/RYO) cigarette consumption among those smoking FM/RYO cigarettes, and prevalence of current smoking among 16–24-year-olds. Time-series analyses were conducted using Autoregressive Integrated Moving Average with Exogenous variables (ARIMAX) regression models including a gradual level change starting in June 2017 and ending in May 2018 for cigarette consumption and a step change in June 2016 for prevalence of current smoking.

**Results:**

The ARIMAX model was not able to detect a change in mean daily cigarette consumption—for FM (B_adj_=−0.543, 95% CI −1.381 to 0.296) or RYO (B_adj_=0.002, 95% CI −0.518 to 0.522) following the implementation of standardised packaging. The unadjusted analysis suggested the implementation of standardised packaging was associated with a small (3%) decrease in smoking prevalence among 16–24-year-olds (B_unadj_=−0.031, 95% CI −0.062 to 0.000), but this association was attenuated after adjustment for covariates (B_adj_=−0.010, 95% CI −0.039 to 0.019).

**Conclusions:**

The implementation of standardised packaging of tobacco products was not associated with a meaningful change in the mean number of FM or RYO cigarettes consumed by people smoking in England, suggesting the larger pack size has not had an unintended consequence of substantially increasing cigarette consumption. However, there was also little evidence that the policy substantially reduced smoking among 16–24-year-olds.

**WHAT IS ALREADY KNOWN ON THIS TOPIC**

- Evidence suggests the implementation of standardised packaging may have been associated with a decline in the odds of smoking. However, its impact on cigarette consumption and the uptake of smoking among young adults was unclear.

**WHAT THIS STUDY ADDS**

- The study found no meaningful change in mean daily cigarette consumption among people smoking in England when minimum pack sizes were introduced. This result indicates no evidence of any substantial unintended consequences of the policy change. However, there was also little evidence that the policy reduced the uptake of smoking among young adults.

**HOW THIS STUDY MIGHT AFFECT RESEARCH, PRACTICE OR POLICY**

- Taking these results together with those of a previous study, it appears that standardised packaging (including minimum pack sizes) may have accelerated the decline in national smoking prevalence among 16–24-year-olds without inadvertently increasing cigarette consumption among people who smoke.

## Introduction

 In May 2016, the UK implemented Standardised Packaging of Tobacco Products Regulations requiring cigarette sticks and packs to have a standardised ‘plain’ appearance and to be sold in minimum quantities of 20 cigarette sticks or 30 g loose tobacco per pack.^1^ The standardised packaging requirements included no specific branding, packaging in drab dark brown colour, combined health warning covering 65% of the package, no misleading information on tar, nicotine or carbon monoxide and no flavour descriptors.^1^ The aims of the legislation were to remove all promotional branding characteristics from the packaging and to increase upfront costs.^2^,^4^ It was particularly intended to stop young people from taking up tobacco smoking.^4^ However, the legislation could have unintentionally caused increased cigarette consumption among people already smoking due to the smaller pack sizes no longer being available. A similar phenomenon has been observed with increased portion sizes for food products.^5^ Therefore, this study aimed to investigate the effects of the legislation on intended and potential unintended consequences.

Tobacco manufacturers were given 1 year from May 2016 until May 2017 to adjust to the new packaging legislation. Evans-Reeves *et al*^6^ reported that most switches from branded to standardised packaging in shops occurred between January and April 2017, showing that the industry took advantage of the 1-year transition period. They also identified other tactics used by the tobacco companies prior to the full implementation in May 2017, such as focussing on the low-price segments, for example, by offering more roll-your-own (RYO) variants, and offering new pack sizes with 23 or 24 factory-made (FM) cigarette sticks.^6^

Opazo Breton *et al*^7^ found the start of the 1-year implementation period was associated with a significant step decrease in the odds of smoking in England. They hypothesised that the reduction in smoking prevalence was induced by the announcement of the standardised packaging rather than the actual switch. Blackwell *et al*^8^ argued that observational evidence suggests that greater packs will lead to greater cigarette consumption. Lee *et al*^9^ conducted a randomised cross-over trial in which they asked participants to buy their usual brand of cigarettes in packs of either 20 or 25 sticks for each 14-day periods. Participants smoked less when purchasing packs of 20 cigarettes compared with the larger sizes. Based on data from a representative UK consumer panel conducted before the minimum pack size regulation took effect, around 68% of FM cigarette pack sizes purchased included between 11 and 19 sticks, and roughly 13% contained 10 sticks.^10^ These data indicate that most people smoking FM cigarettes would have had to switch to larger pack sizes after the policy implementation. In contrast, the effect on RYO tobacco purchases was less pronounced. Prior to the regulation, around 60% of RYO purchases were already packs containing more than 30 g, and 12.5 g to 29 g and <12.5 g pack sizes were at only about 15% each.^10^

It is important to investigate whether the introduction of minimum pack sizes has led to an increase in daily cigarette consumption among people smoking cigarettes given the direct and indirect health benefits of smoking reduction. In a meta-analysis, Chang *et al*^11^ found that reducing cigarette consumption from heavy to moderate, or by at least 50%, significantly decreased lung cancer risk. Further, changing from heavy to light smoking significantly reduced cardiovascular disease risk. The indirect effect of reduced cigarette consumption is that it can help people quit smoking.^12 13^ Therefore, if the introduction of minimum pack sizes, in fact, led to an increase in daily cigarette consumption among people smoking cigarettes, this unintended negative consequence would need to be weighed against the potential benefit of the policy in reducing smoking uptake among young people.

This study aimed to investigate both potential consequences of the standardised packaging policies in England: whether the implementation of regulations resulted in a change in mean daily cigarette consumption among people smoking cigarettes and whether it led to a decline in smoking uptake among young adults. As there is reason to believe that the legislation may have had different effects on mean daily cigarette consumption for those predominantly smoking FM or RYO cigarettes, respectively, separate analyses were performed for each of these groups. The specific objectives were to assess whether, in England, the introduction of Standardised Packaging of Tobacco Products Regulations—including minimum pack sizes—in May 2017—was associated with (1) a change in mean daily FM cigarette consumption among adults predominantly smoking FM cigarettes; (2) a change in mean daily RYO cigarette consumption among adults predominantly smoking RYO cigarettes; and (3) a decrease in smoking prevalence among 16–24-year-olds.

## Methods

### Study design and participants

This was a population-based, repeat cross-sectional study using data from the Smoking Toolkit Study.^14^ Data were collected face-to-face between November 2007 and January 2020 by a market research company. Each month approximately 1700 adults aged 16 years or older were surveyed in England. To be included, people had to speak English and live in a private household. Households were selected using a hybrid of random location and quota sampling. The locations were randomly selected from roughly 170 000 output areas stratified by a geodemographic classification of the population called Acorn classification from Consolidated Analysis Centers, Inc (CACI). It segments UK households, postcodes and neighbourhoods into 6 categories, 18 groups and 62 types using a combination of social factors and population behaviour.^15^ Interviews were conducted with one member from each household (only one household member can be interviewed) until quotas based on factors influencing the probability of being at home (eg, age, gender, working status) were fulfilled.^14^ Participant identifiable information was removed before the research team accessed the data. The manuscript followed the Strengthening the Reporting of Observational Studies in Epidemiology statement.^16^

### Outcome variables and covariates

The primary outcome measures were mean daily FM/RYO cigarette consumption among people predominantly smoking FM/RYO cigarettes and current smoking prevalence among 16–24-year-olds. Current cigarette smoking was measured with the question ‘Which of the following best applies to you?’. The answer options included ‘I smoke cigarettes (including hand-rolled) every day’, ‘I smoke cigarettes (including hand-rolled), but not every day’, ‘I do not smoke cigarettes at all, but I do smoke tobacco of some kind (eg, Pipe, cigar or shisha)’, ‘I have stopped smoking completely in the last year’, ‘I stopped smoking completely more than a year ago’, ‘I have never been a smoker (ie, smoked for a year or more)’ and ‘Don’t know’. Those who replied with the first or second answer option were classified as currently smoking cigarettes.

Those smoking cigarettes were further asked ‘How many cigarettes do you usually smoke?’. Responses could be provided per day, week or month. In a follow-up question, they were asked ‘How many of these do you think are hand-rolled?’. If they stated that at least 50% of cigarettes smoked were RYO, they were classified as predominantly smoking RYO cigarettes; otherwise, they were classified as predominantly smoking FM cigarettes. For the sensitivity analysis, participants were classified as exclusively smoking RYO cigarettes if 100% of cigarettes smoked were RYO, and as exclusively smoking FM cigarettes if 0% were RYO. Mean daily FM/RYO cigarette consumption was then determined by the number of FM/RYO cigarettes smoked per day among FM/RYO users, respectively.

Current smoking among 16–24-year-olds was determined with the same question as for current cigarette smoking, but with the additional answer option ‘I do not smoke cigarettes at all, but I do smoke tobacco of some kind (eg, pipe or cigar)’. The number of 16–24-year-olds classified as currently smoking was divided by the total number of 16–24-year-olds. For the sensitivity analyses, the number of those aged 25 and over classified as currently smoking was divided by the total number of participants in this age group.

Time was measured in months, representing the monthly survey wave, and was coded from 1 (November 2007) to 147 (January 2020). Covariates included a composite score for other tobacco control policies, tobacco tax increases and mass media expenditure (aggregated at the country level). In addition, analyses of smoking prevalence among 16–24-year-olds also adjusted for (1) prevalence of prescription medication use for smoking cessation, (2) prevalence of over-the-counter nicotine replacement therapy use or face-to-face behavioural support for smoking cessation and (3) prevalence of e-cigarette use. These estimates were aggregated across England for each month. Even though we used current smoking prevalence among 16–24-year-olds as a proxy for smoking uptake, we do not consider these covariates to be predictors of uptake. Rather, we included these in the model as covariates because they are related to smoking cessation. By measuring current smoking prevalence rather than uptake, the prevalence data can be confounded by people quitting smoking. Detailed descriptions of the covariates and numbers of missing values for each collected variable required for the analysis are reported in the online supplemental table S1.

### Analysis

The analysis was conducted in RStudio (V.2022.07.2, R V.4.2.1). The level of significance was set to 0.05 and 95% CIs are provided. Data were aggregated monthly and weighted using raking^17^ to match the population of England based on sociodemographic characteristics. Data were analysed using Autoregressive Integrated Moving Average with Exogenous variables (ARIMAX) regression models using the TSA package in R,^18^ following recommended procedures for time-series analysis.^19 20^ Unadjusted and adjusted models are reported. Details of the methods and the model selection process are provided in the online supplemental file 1 and the pre-registered protocol (https://osf.io/uaxc8/).

For the first and second research questions (associations with cigarette consumption), the effects of the intervention (ie, standardised packaging) were modelled using a gradual change, coded 0 up to May 2017, then increasing by 1/12 each month, eventually approaching the long-term level change of 1 from June 2018 onwards. The gradual level shift was included in the model as a transfer function. The adjusted model included the composite tobacco control score as a dummy variable and mass media expenditure as a transfer function. We conducted three sensitivity analyses. First, we modelled the effect of the intervention as a step change in June 2017 (coded 0 up to May 2017, and 1 from then onwards). Second, we modelled the effect of the intervention as a gradual level shift from June 2016 until May 2017 (ie, a year earlier than our primary analysis; coded 0 up to May 2016, then increasing by 1/12 until May 2017 and coded 1 from then onwards) because, even though our primary hypothesis was that the policy took effect once the grace period for tobacco manufacturers had past and it was fully implemented, we additionally wanted to test a potential effect starting at the time the policy was first implemented (beginning of the grace period), as was found by research assessing the policy’s impact on smoking prevalence.^7^

Third, we modelled trends in mean daily cigarette consumption for those exclusively (rather than predominantly, as in our primary analysis) smoking FM and RYO cigarettes, respectively. Finally, we calculated Bayes factors (using an online calculator,^21^ results presented in the online supplemental file) to test whether there was evidence for the null hypothesis of no difference or if the data were insensitive to detect an effect corresponding to an increase in mean daily cigarette consumption.^22^,^24^ Alternative hypotheses were represented using half-normal distributions and the expected effect size for a post-intervention change in the trend for mean daily cigarette consumption were set for medium (B=0.2) effect sizes based on previous research.^25^

For the third research question (association with smoking among 16–24-year-olds), the effect of the intervention was modelled using a step change, coded 0 up to May 2016, and 1 from then onwards. We used a diverging approach for this research question because we assumed that the introduction of Standardised Packaging of Tobacco Products Regulations may have been more likely to cause a step change in the decline in the rate of current smoking among 16–24-year-olds at the beginning of the introduction than a gradual change. This approach is in line with findings from Opazo Breton *et al*^7^ who found a significant change in the level but not the slope of the odds of smoking, starting after the introduction of the policy in May 2016.

The adjusted model included the composite tobacco control score as a dummy variable and mass media expenditure, prevalence of prescription medication use for smoking cessation, the prevalence of use of nictotine replacement therapy over the counter or face-to-face behavioural support for smoking cessation, and prevalence of e-cigarette use as transfer functions. As sensitivity analyses, a step change in June 2017 and a gradual level shift from June 2016 until May 2017 (ie, the level shift proposed for the primary analysis assessing changes in mean daily cigarette consumption) were modelled. Additionally, adults 25 years and older served as a control group to assess whether the final model selected for smoking among 16–24-year-olds measured an effect specific to 16–24-year-olds (ie, smoking uptake among young people) by testing the final model in the older age group.

### Pre-registration

Our study protocol was pre-registered on Open Science Framework (https://osf.io/uaxc8/). We made two amendments. First, we stated that we would include tobacco tax increases for FM and RYO cigarettes separately. Because RYO cigarette tax increases occurred at the same time as those on FM cigarettes, the two variables were highly correlated and were therefore combined into one variable. Second, we stated that we would decide whether differencing was required to make the time-series stationary based on the results of the Augmented Dickey-Fuller test. However, as the visual inspection of the time-series plots suggested differencing was required, we decided to give more weight to the visual inspection than the test results (which indicated no differencing; details in online supplemental file 1). We used 1-lag differencing in the main analyses and performed sensitivity analyses without differencing.

We undertook several unplanned sensitivity analyses. We modelled mean daily FM cigarette consumption with zero lags for each covariate because the cross-correlation function plots did not show a strong correlation between mean daily FM cigarette consumption and the respective covariates. We also included models assessing the potential effect of standardised packaging on mean daily FM or RYO cigarette consumption, respectively, among all people smoking cigarettes (rather than only those predominantly smoking FM/RYO cigarettes).

## Results

Data were collected from 257 929 participants (unweighted).^26^ Of these, 51 063 (19.8%) participants smoked cigarettes: 20 487 (40.1%) predominantly smoked RYO cigarettes and 29 188 (57.2%) predominantly smoked FM cigarettes (unweighted, 1388 with missing data on the type of cigarette predominantly smoked). Among 37 158 participants aged 16–24 years, 9366 (25.2%) reported current smoking (unweighted). The sociodemographic characteristics of study participants are presented in online supplemental table S3.

Across the entire study period (November 2007 to January 2020), people predominantly smoking FM cigarettes consumed, on average, 11.2 (95% CI 11.1 to 11.4) FM cigarettes per day. The mean consumption was 10.9 (95% CI 9.7 to 12.1) FM cigarettes per day just before the policy started in May 2016 and 10.4 (95% CI 9.0 to 11.7) 1 year after the full implementation in May 2018. Among people predominantly smoking RYO cigarettes, the mean daily consumption across the entire study period was 11.8 (95% CI 11.6 to 12.0) RYO cigarettes. In May 2016, the mean consumption was 12.0 (95% CI 10.7 to 13.3) RYO cigarettes per day and, in May 2018, 10.9 (95% CI 9.7 to 12.1) RYO cigarettes per day. Across the entire study period, the mean prevalence of current smoking among 16–24-year-olds was 24.4% (95% CI 23.8 to 25.1). In May 2016, the prevalence of current smoking among 16–24-year-olds was 27.7% (95% CI 22.1 to 33.4) and, in May 2018, 24.5% (95% CI 19.0 to 29.9), respectively.

### Mean daily FM cigarette consumption

**Figure 1:**
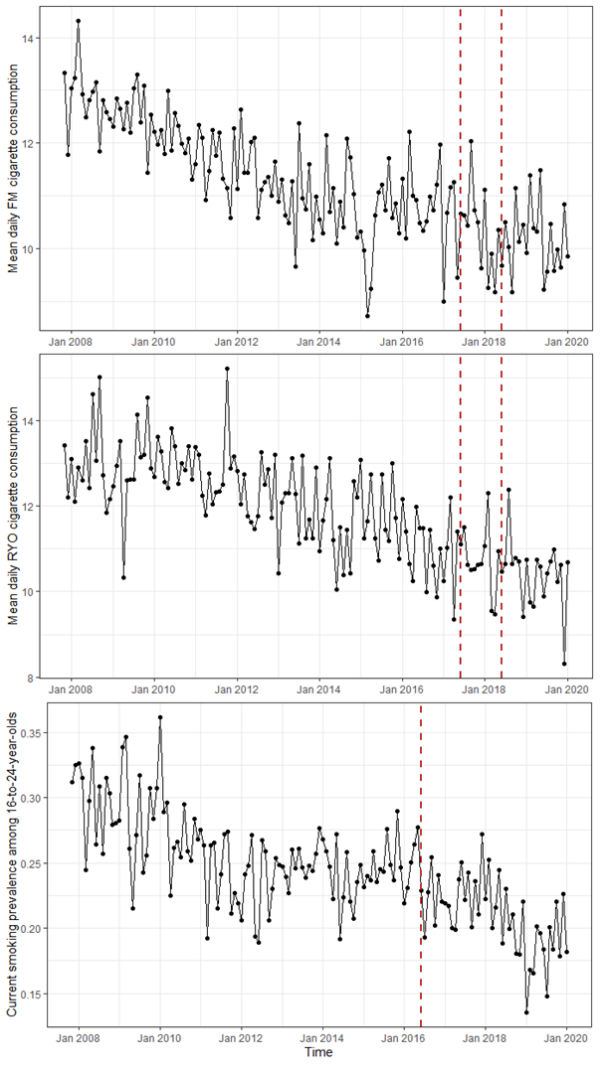
Time series for mean daily FM cigarette consumption among people smoking predominantly FM cigarettes (top), mean daily RYO cigarette consumption among people smoking predominantly RYO cigarettes (middle) and current smoking prevalence among 16–24-year-olds (bottom). The red lines indicate the hypothesised period of the policy effect (which we modelled as a gradual level change for mean daily FM/RYO cigarette consumption and a step change for current smoking prevalence among 16–24-year-olds). FM, factory-made; RYO, roll-your-own.

Overall, mean daily FM cigarette consumption among those predominantly smoking FM cigarettes decreased between November 2007 and January 2020, especially until approximately 2015, after which it appears to have stagnated (figure 1). The implementation of standardised packaging, modelled as a gradual level shift, was not associated with a significant change in mean daily FM cigarette consumption (unadjusted B=−0.681, 95% CI −1.584 to 0.222; adjusted B=−0.543, 95% CI −1.381 to 0.296; table 1, full model specifications in online supplemental table S6). Similarly, in all but one of the sensitivity analyses, there was no significant association between the implementation of standardised packaging and mean daily FM cigarette consumption (online supplemental tables S7–S12). A significant association was found in the model without differencing, but this model did not fit the data well.

**Table 1:** Results for best fitting models for the association of implementation of standardised packaging with the three different outcomes—unadjusted and adjusted for covariates

Outcome	Modelled intervention effect	Model specification	Unadjusted		Adjusted*	
			B (95% CI)	P value	B (95% CI)	P value
Mean daily FM cigarette consumption	Gradual level change, from June 2017 to May 2018	ARIMAX (0,1,1)	−0.681 (−1.584 to 0.222)	0.139	−0.543 (−1.381 to 0.296)	0.204
Mean daily RYO cigarette consumption		ARIMAX (0,1,1)	−0.473 (−1.472 to 0.527)	0.354	0.002 (−0.518 to 0.522)	0.994
Current smoking prevalence among 16–24-year-olds	Step change, from June 2016	ARIMAX (0,1,1)	−0.031 (−0.062 to 0.000)	0.047	−0.010 (−0.039 to 0.019)	0.497

* Adjusted for tobacco control policies, tax increases, mass media expenditure and for current smoking among 16–24-year-olds additionally use of prescription medication in quit attempts, use of over-the-counter medication or behavioural support in quit attempts and e-cigarette use. For full model specifications, see supplementary Tables S6, S15, and S23online supplemental tables S6, S15, and S23.

ARIMAX, Autoregressive Integrated Moving Average with Exogenous variables; FM, factory-made; RYO, roll-your-own.

### Mean daily RYO cigarette consumption

As seen for FM cigarettes, the mean daily RYO cigarette consumption among people predominantly smoking RYO cigarettes decreased throughout the study period, particularly up to around 2015, remaining relatively stable thereafter (figure 1). The implementation of standardised packaging seems not to have led to a significant change in this trend. The modelled gradual level shift was not significantly associated with mean daily RYO cigarette consumption in the unadjusted (B=−0.473, 95% CI −1.472 to 0.527) or the adjusted model (B=0.002, 95% CI −0.518 to 0.522; table 1, full model specifications in online supplemental table S15). All but one sensitivity analyses also did not show a significant change in mean daily RYO cigarette consumption (online supplemental tables S16–S20). The exception was the model without differencing. However, this model did not fit the data well.

### Current smoking among 16–24-year-olds

The prevalence of current smoking among 16–24-year-olds decreased over time (figure 1). In the unadjusted model, the standardised packaging implementation was associated with a small decline in current smoking prevalence among 16–24-year-olds (B=−0.031, 95% CI −0.062 to 0.000), but in the adjusted analysis, the association was attenuated and not significant (B=−0.010, 95% CI −0.039 to 0.019; table 1, full model specifications in online supplemental table S23). The result was comparable when using a model without differencing (online supplemental table S24), but the model did not fit the data well. The remaining sensitivity analyses resulted in non-significant Beta coefficients for the standardised packaging implementation in the unadjusted and adjusted models, with the Beta coefficients all being close to zero (online supplemental tables S25 and S26). In the analysis assessing current smoking among those aged 25 years and over, using the same model as for those aged 16–24 years, the unadjusted model showed significant autocorrelation suggesting that the model did not fit the data of the control group well (therefore, the adjusted model was not further considered, but presented for completeness; online supplemental figures S14, S15 and table S25).

## Discussion

### Summary

The results suggest that the data were insensitive to detect a true association between the intervention (ie, standardised packaging, modelled as a gradual level shift) and mean daily cigarette consumption. This applied to both FM and RYO cigarettes. For uptake among young people, modelled using current smoking prevalence among 16–24-year-olds, the unadjusted analysis showed a significant negative association with the modelled step change in June 2016. The result indicates that the introduction of standardised packaging led to a decrease in the mean prevalence of 16–24-year-olds currently smoking by 3% (~0.8 percentage point decline). However, when adjusting for covariates, the association was no longer significant, suggesting that the policy may have been confounded with changes in one of the covariates, such as mass media expenditure or tax increases. In summary, our results provide no evidence of substantial unintended consequences of increased cigarette consumption due to the implementation of standardised packaging.

### Comparison with previous studies

There have been long-term declines in mean cigarette consumption among people smoking in England,^27 28^ and we observed a similar decline up to about 2015. Our data showed, more recently, mean cigarette consumption no longer appears to be declining in England, which may have contributed to concerns about whether the change in pack size was responsible. However, these fine-grained and formal analyses did not detect a significant association between the implementation of standardised packaging and cigarette consumption among people smoking, which may indicate that the long-term decline stopped before the policy change. Visual inspection of the data suggests a long-term decline in mean consumption up to approximately 2015, followed by a period where the decline appears to have ceased. Although efforts were made to select ARIMAX specifications that account for the observed trend, it is important to recognise that the modelling approach may not fully address the complexities of the changing behaviour in the time series data. A recent study using the same data showed that declines in FM consumption happened predominantly between 2008 and 2015 and increases in RYO consumption started slowing down notably from early 2016 onwards.^29^

Previous research assessing the impact of the implementation of standardised packaging on smoking prevalence in England found a significant step reduction in the odds of smoking.^7^ However, the study did not measure whether the observed effect was due to quitting or a reduction in uptake, nor did it evaluate the impact of the policy on smoking behaviour among people continuing to smoke. The study reported that in the age group of 18–25-year-olds there was a significant level change after May 2017 in the odds of smoking after adjusting for covariates (OR 1.29, 95% CI 1.03 to 1.62).^7^ When comparing this result to ours, it could be interpreted that the implementation of standardised packaging might have led to a reduction in smoking prevalence among young people, but that the reduction is not attributable to reduced uptake because the effect we observed in young people was reduced after adjusting for covariates, including those related to quitting behaviour.

Research from Australia and Norway evaluated the implementation of standardised packaging but crucially the policies under study did not entail the introduction of minimum pack sizes. The introduction of plain packaging in Australia did not have a significant impact on smoking prevalence compared with New Zealand where this legislation was not implemented at the same time.^30^ The Australian data also indicated that plain packaging may have led to an increase in the number of cigarettes consumed per week. However, this result was only significant at a 0.1 level.^30^ Data from Norway suggested no significant change in smoking prevalence due to standardised packaging, but the results were overall inconclusive, and it is possible that the policy accelerated the declining trend in smoking prevalence that was already taking place at the time.^31^ The study did not assess the impact of the policy on cigarette consumption.

### Strengths and limitations

This study is the first to investigate the impact of standardised packaging on mean daily cigarette consumption in England. A strength of the study is that it used data from a representative population-based survey. Previous research has shown that the self-reported mean daily cigarette consumption from this survey is comparable to sales data.^32^ Among the limitations are that only data until January 2020 were used for the analysis, which is less than 3 years after the full implementation of the intervention, which may have contributed to the issues with data insensitivity. This cut-off was chosen because after January 2020 the COVID-19 pandemic caused several disruptions, including changes in data collection and changes in smoking behaviour in the population.^33^

Another limitation is that it is difficult to separate the potential effect of this one specific legislation from all the other changes that occurred at a similar time (eg, tobacco tax increases, changes in mass media expenditure), and also to isolate the effects of individual components of the legislation from the remaining (eg, plain packaging vs minimum pack size). Previous research found that large pictorial health warnings on tobacco packaging can improve health knowledge and risk perception, thereby promoting smoking cessation.^3^ Another study reported that the introduction of the partial tobacco point-of-sale display ban in England in 2012 was followed by a decline in the trend of smoking prevalence.^25^ The third tobacco control policy that was introduced during the study period was a complete point-of-sale ban which may contribute to a change in smoking perceptions and a decrease in impulse tobacco purchasing.^34^ Due to these rapid changes in the tobacco control climate during the study period as well as the staggered implementation of the plain packaging legislation, impacts may have occurred at different times in different locations. We therefore did not include a model of the counterfactual scenario in the analysis, as this may oversimplify the intervention’s impact and failing to capture these diverse effects could lead to an incomplete understanding of the intervention’s true impact. Nonetheless, our findings do not show any unintended effect of this legislation (collectively) on cigarette consumption.

Further, we were only able to assess current smoking prevalence among young people (aged 16–24 years) as a proxy for smoking uptake instead of directly measuring smoking initiation due to the cross-sectional nature of the survey data. Current smoking prevalence was defined as smoking tobacco products either daily or non-daily which is an approach previously used by other studies using the same data set as well as other youth surveys in the UK (eg, YouGov commissioned Action on Smoking and Health and the Annual Government Survey).^35^ However, there was no criterion for a minimum number of lifetime cigarettes as often included in a US context.^36^ Lastly, it is theoretically possible that participants were selected more than once from the English population over time, but the chances are very small. As the research team only has access to the deidentified data, it was not possible to check whether that was the case.

## Conclusions

This study could not detect a meaningful change in mean daily FM and RYO cigarette consumption in England attributable to the implementation of standardised packaging, including the minimum pack size legislation. There was an indication for a slight decrease in current cigarette smoking among 16–24-year-olds due to the implementation of standardised packaging, but this was not significant when adjusting for covariates. Overall, the data did not provide any evidence of substantial unintended consequences of the policy change.

## Supplementary material

10.1136/tc-2023-058560 online supplemental file 1

## Data Availability

Data are available in a public, open access repository.
