# Supplementary material for: Impact of Standardised Packaging of Tobacco Products Regulations on cigarette consumption and youth smoking in England: interrupted time-series analysis
Source: Tob Control. 2024 Jun 8;34(5):e058560. doi: 10.1136/tc-2023-058560 (PMC12573331; doi:10.1136/tc-2023-058560)
Supplement: online supplemental file 1 [file tc-34-5-s001.pdf]

SUPPLEMENT

Article: “Impact of Standardised Packaging of Tobacco Products Regulations on cigarette consumption and youth smoking in England: interrupted time-series analysis”

Contents

Methods 2
- Covariates 2
- Missing values and outliers 3
- Determining trends and stationarity 4
- General note regarding the interpretation of the fitted ARIMAX models 4
- R Packages (including their dependencies) used for analyses 4

Sociodemographic characteristics of study participants 5

Results for mean daily factory made (FM) cigarette conumption 6
- Sensitivity analysis 1a: ARIMAX(0,1,1) with lag 0 for all covariates 9
- Sensitivity analysis 1b: ARIMAX(0,0,1) 9
- Sensitivity analysis 1c: alternative gradual level shift 10
- Sensitivity analysis 1d: alternative step change 10
- Sensitivity analysis 1e: exclusive FM cigarette consumption 11
- Sensitivity analysis 1f: FM cigarette consumption among all who smoke cigarettes 11

Results for mean daily roll-your-own (RYO) cigarette consumption 12
- Sensitivity analysis 2a: ARIMAX(0,0,1) 15
- Sensitivity analysis 2b: alternative gradual level shift 15
- Sensitivity analysis 2c: alternative step change 16
- Sensitivity analysis 2d: exclusive RYO cigarette consumption 16
- Sensitivity analysis 2e: RYO cigarette consumption among all who smoke cigarettes 17

Results for current smoking among 16-to-24-year-olds 18
- Sensitivity analysis 3a: ARIMAX(0,0,1) 22
- Sensitivity analysis 3b: alternative step change 23
- Sensitivity analysis 3c: alternative gradual level change 23
- Sensitivity analysis 3d: current smoking among those aged 25 years and older 24

References 27

Methods

Covariates

A composite score was created to account for other tobacco control policies that were implemented during the study period (i.e., pictorial health warnings on product packaging in October 2008, supermarket point-of-sale display ban in April 2012, full point-of-sale ban in April 2015). The dummy variable was coded as 1 in October 2008, April 2012, and April 2015, and 0 in all other months. For each of these policies, studies found that they are positively associated with quitting-related outcomes at the population-level [1-3].

Another variable accounted for tobacco tax increases starting with 1 in November 2007 and increasing proportionally to the implemented tax increases for FM and RYO cigarettes in the months they were implemented. Tax increases on RYO cigarettes were always implemented simultaneously with tax increases on FM cigarettes. As the increases for RYO were greater, these values were used rather than the ones for FM cigarettes. A previous study showed that above inflation tax increases implemented between March 2010 and December 2019 were inconsistently associated with temporary declines in smoking prevalence, per-capita self-reported cigarette consumption, and higher quit success rates [4].

Further, quarterly mass media expenditure (in million £) data aggregated at the country-level were obtained from the UK Office of Health Improvement and Disparities on campaigns (including ‘Smokefree’, ‘Stoptober’, and ‘Health Harms’) in TV, radio, print, cinema, and online for each month. Previous research found that higher expenditure on tobacco control mass media was associated with higher success rates of quitting [5] and, conversely, that a suspension of tobacco control mass media campaigns was associated with a decrease in quit line calls, visits to the national smoking cessation website and requests for cessation support packs [6].

Among 16-to-24-year-olds, additionally, (i) prevalence of prescription medication use for smoking cessation, (ii) prevalence of over-the-counter nicotine replacement therapy use or face-to-face behavioural support for smoking cessation, and (iii) prevalence of e-cigarette use were calculated by dividing the number of respondents aged 16 to 24 years who reported (i) having used nicotine replacement therapy on prescription, bupropion, or varenicline during their most recent serious quit attempt within the past year, (ii) having used NRT over the counter or face-to-face behavioural support during their most recent serious quit attempt within the past year, and (iii) currently using e-cigarettes, respectively, by the total number of respondents aged 16 to 24 years. People were classified as using e-cigarettes if they answered “electronic cigarette/e-cigarette” or “Juul” to any of the following three questions: “Which, if any, of the following are you currently using to help you cut down the amount you smoke?”, “Can I check, are you using any of the following either to help you stop smoking, to help you cut down or for any other reason at all?”, or “Do you regularly use any of

the following in situations when you are not allowed to smoke?”. For the sensitivity analysis, the three covariates specific to 16-to-24-year-olds were additionally computed for those aged 25 years and older. All these estimates were aggregated across England.

Missing values and outliers

In March 2013, two waves of data were collected which were averaged for the analysis. In December 2008, no data were collected. For this month, data were imputed by using the average of the previous and subsequent month. The number of missing values for each variable is reported in Table S1. For the outcome of mean daily cigarettes, values of over 80 cigarettes smoked per day were considered outliers and excluded from the analysis [7]. This was the case for 17 participants. Further, boxplots were created for all relevant variables and the ‘tsoutliers’ function from the ‘forecast’ package by Hyndman and Khandakar was used to detect potential outliers [8]. Identified outliers and replacement values are presented in the supplement (Table S2).

Table S1: Variables used for the analysis and the number (%) of missing values for each of them.

Variable	Missing values n (%)
Age	1006 (0.4)
Smoking status	188 (0.1)
Cigarette consumption among those currently smoking cigarettes (n = 52331)	1268 (2.4)
Proportion of roll-your-own among those currently smoking cigarettes (n = 52331)	2656 (5.1)
Use of smoking cessation aid among those with serious quit attempt in last year (n = 18834)	813 (4.3)

Table S2: Variables with outliers detected by ‘tsoutliers’-function and their replacement values.

Variable	Month(s) of data collection	Outlier value(s)	Replacement value(s)
Use of smoking cessation aid in last serious attempt – prescription medication (16-to-24-year-olds), proportion	July 2008	0.1154901	0.03181801
Use of smoking cessation aid in last serious attempt – over-the-counter medication or behavioural support (16-to-24-year-olds), proportion	February 2009	0.1006351	0.04945777
E-cigarette use (16-to-24-year-olds), proportion	November 2016	0.09161903	0.03740511
E-cigarette use (25 years and older), proportion	July 2013,<br>January 2014,<br>August 2014,<br>June 2015,<br>September 2015,<br>July 2016,<br>October 2016,<br>March 2017,<br>July 2018,<br>October 2019	0.04871597<br>0.02778039<br>0.05386517<br>0.05648386<br>0.05914551<br>0.05899493<br>0.02994447<br>0.02856382<br>0.04738789<br>0.01946706	0.03783663<br>0.05024255<br>0.04354962<br>0.04479617<br>0.04707607<br>0.03962700<br>0.04194363<br>0.03725602<br>0.03326595<br>0.03058441

Determining trends and stationarity

We visually inspected the plotted time series and used unit root tests to determine whether non-seasonal or seasonal differencing was required for stationarity. For the visual inspection, we plotted each time series and additionally decomposed them into three components: a trend, a seasonal, and a random component. We inspected the plots for signs of non-stationarity by looking for evidence of a trend in the mean, variance, autocorrelation, or seasonality [9, 10]. Additionally, we used the Augmented Dickey-Fuller test [11] (ndiffs function in R) to check for the number of non-seasonal differences and the seasonal unit root test according to Wang, Smith & Hyndman [12] (nsdiffs function in R) to check the number of seasonal differences required to make the time series stationary. The unit root tests have a bias towards the unit root alternative (i.e., no differencing required) [10]. Therefore, we decided to give more weight to the visual inspection of the time series.

General note regarding the interpretation of the fitted ARIMAX models

The AR term captures the relationship between an observation in the time series and a certain number of lagged observations. A positive AR coefficient (ϕ) indicates that past values of the time series have a positive linear relationship with the current value. In other words, if ϕ is positive, an increase in past values leads to an increase in the current value. Conversely, a negative AR coefficient suggests an inverse relationship, where an increase in past values leads to a decrease in the current value. The order of the AR term (p) indicates the number of lagged observations considered in the model. The MA term captures the relationship between the current value of the time series and past forecast errors (residuals). A positive MA coefficient (θ) indicates that past forecast errors have a positive linear relationship with the current value. In other words, if θ is positive, an increase in past forecast errors leads to an increase in the current value. Similarly, a negative MA coefficient suggests an inverse relationship, where an increase in past forecast errors leads to a decrease in the current value. The order of the MA term (q) indicates the number of past forecast errors considered in the model.

R Packages (including their dependencies) used for analyses

- tidyverse [13]
- survey [14]
- tibbletime [15]
- forecast [16]
- ggplot2 [17]
- tseries [18]
- TSA [19]

Sociodemographic characteristics of study participants

Table S3: Sociodemographic characteristics of study participants by years of data collection.

Characteristic	Years of data collection			
	**2007-2010**	**2011-2013**	**2014-2016**	**2017-2020**
Age, median (IQR)	44 (30, 61)	45 (30, 61)	46 (31, 62)	46 (31, 62)
Women, n (%, 95% CI)	35,157 (51.4, 51.0-51.8)	33,489 (51.2, 50.8-51.6)	30,940 (51.0, 50.6-51.4)	31,026 (48.9, 48.5-49.3)
Men, n (%, 95% CI)	33,232 (48.6, 48.2-49.0)	31,955 (48.8, 48.4, 49.2)	29,272 (49.0, 48.6-49.4)	31,026 (51.0, 50.6-51.4)
Non-binary[1], n (%, 95% CI)	NA	NA	NA	60 (0.1, 0.1-0.1)
Social grade AB, n (%, 95% CI)	18,384 (26.9, 26.5-27.3)	17,826 (27.2, 26.8-27.6)	16,402 (27.0, 26.6-27.4)	17,268 (27.2, 26.8-27.6)
Social grade C1, n (%, 95% CI)	20,010 (29.3, 28.9-29.6)	18,658 (28.5, 28.1-28.9)	16,641 (27.4, 27.1-27.8)	17,840 (28.1-27.8-28.5)
Social grade C2, n (%, 95% CI)	14,016 (20.5, 20.2-20.8)	13,989 (21.4, 21.0-21.7)	13,335 (22.0, 21.6-22.3)	13,147 (20.7, 20.4-21.1)
Social grade D, n (%, 95% CI)	10,534 (15.4, 15.1-15.7)	9,834 (15.0, 14.7-15.3)	9,138 (15.1, 14.8-15.4)	9,406 (14.8, 14.5-15.2)
Social grade E, n (%, 95% CI)	5,444 (8.0, 7.8-8.1)	5,138 (7.9, 7.7-8.0)	5,150 (8.5, 8.3-8.7)	5,778 (9.1, 8.9-9.4)
Cigarette smoking, n (%, 95% CI)	14,814 (21.7, 21.3-22.0)	13,086 (20.0, 19.7-20.3)	11,168 (18.4, 18.1-18.8)	10,509 (16.6, 16.3-16.9)

[1]Non-binary was introduced as an answer option in May 2017.

[2]Occupational social grades are a measure of socioeconomic position, with AB being the most and E the least socioeconomically advantaged [20].

Results for mean daily factory made (FM) cigarette conumption

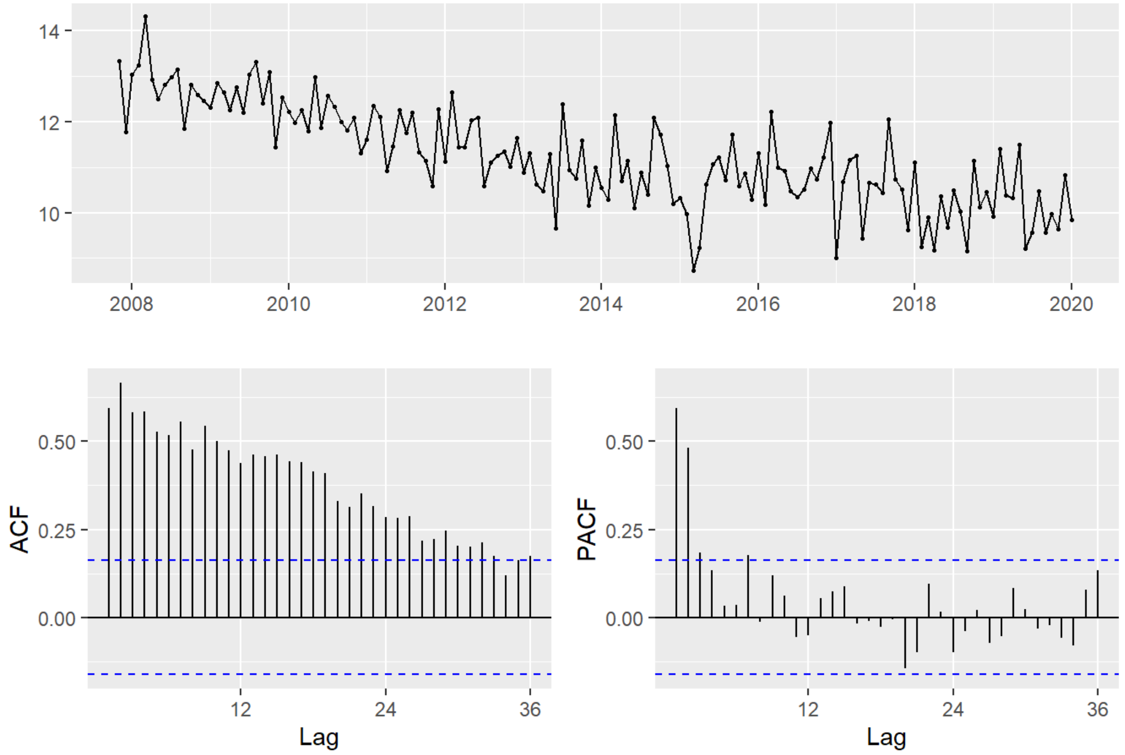

Figure S1: Time series, auto-correlation function (ACF), and partial auto-correlation function (PACF) graphs of mean daily FM cigarette consumption as the outcome variable.

- Augmented Dickey-Fuller test shows no differences required to make series stationary.
- As time series in Figure S1 indicates that differencing might be required, use lag-1 difference in main analysis with further sensitivity analysis without lag-1 difference.
- Seasonal unit root test shows no seasonal differences required to make series seasonally stationary.

Table S4: Akaike information criterion (AIC) for different ARIMAX models for mean daily FM cigarette consumption as the outcome variable (unadjusted).

ARIMAX	AIC
(1,1,0)	345.8
(0,1,1)	**315.4**
(1,1,1)	316.0
(2,1,0)	337.8
(0,1,2)	316.3
(2,1,1)	316.6
(1,1,2)	316.8

➔ Smallest AIC for ARIMAX(0,1,1), choose this model

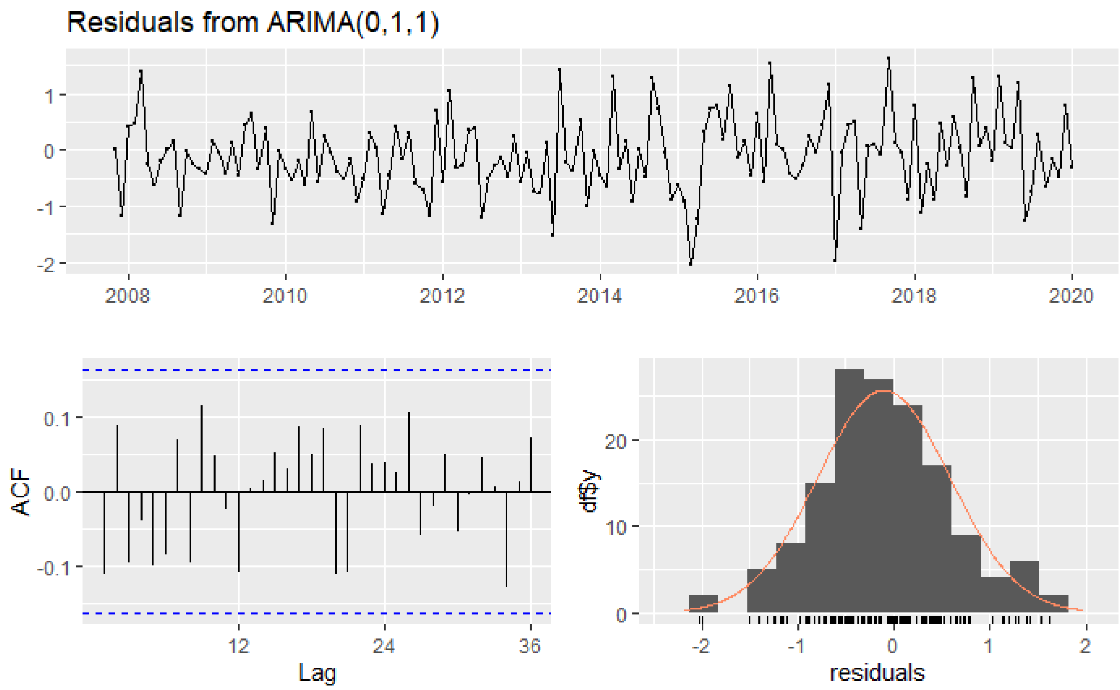

Figure S2: Residuals time series plot, residuals auto-correlation function (ACF) plot, and residuals histogram for unadjusted ARIMAX(0,1,1) model for the outcome of mean daily FM cigarette consumption.

- Ljung-Box test results for residuals from ARIMAX(0,1,1) for the outcome of mean daily FM cigarette consumption with non-zero mean: Q*=23.39, df=23, p=0.438 → no significant autocorrelation.

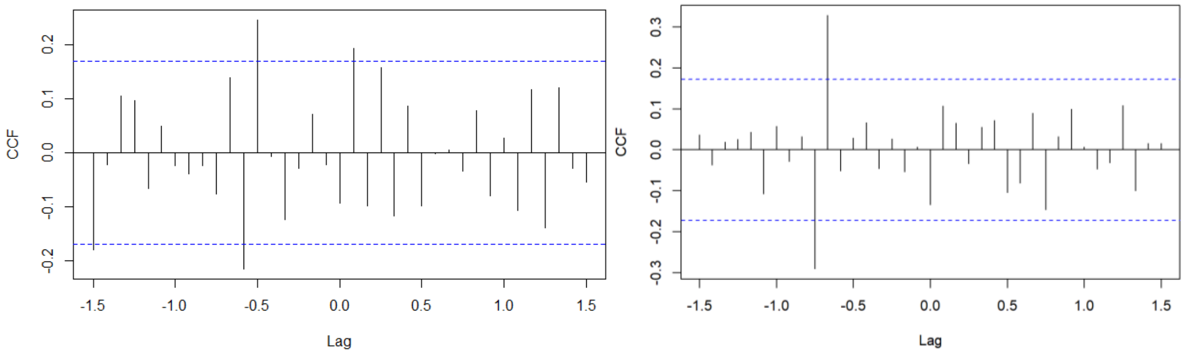

Figure S3: Cross correlation function plot for the mean daily FM cigarette consumption time series and (1) tax increase time series and (2) mass media expenditure time series (from left to right).

Table S5: AIC values for mass media included as transfer function in the ARIMAX(0,1,1) model for the outcome of mean daily FM cigarette consumption, using different numbers of AR terms.

AR terms	Tax increase, AIC	Mass media expenditure, AIC
0	316.9	317.5
1	318.4	318.9
2	316.1	**316.3**
3	**304.0**	317.1

- For tax increases, smallest AIC with 3 lags, and still the smallest when fixing lag 1 and lag 2 to zero and just testing for lag 3 (AIC=304.0); hence, go with 3 lags for tax increases.
- For mass media expenditure, smallest AIC with 2 lags, but less than 2 units difference to 0 lags; so, go with 0 lags.

- Adjusted model: ARIMAX(0,1,1) with dummy variable for tobacco control policies, transfer function for tax increases (3,0), and transfer function for mass media expenditure (0,0) using a gradual level change to model the intervention effect of standardised packaging.

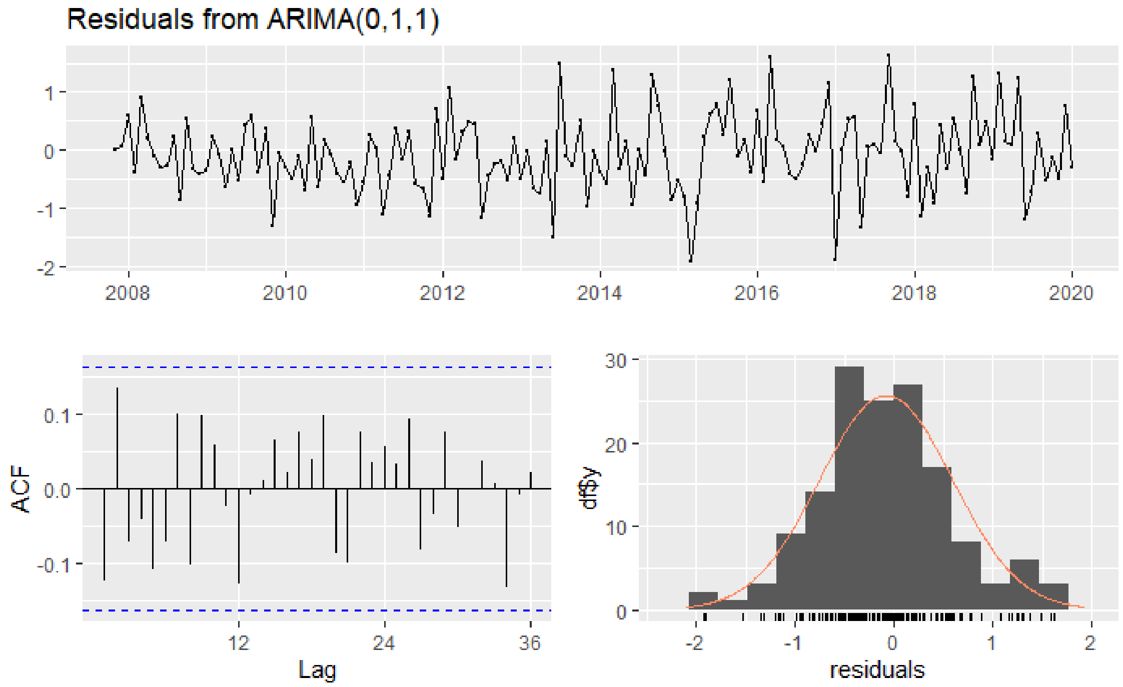

Figure S4: Residuals time series plot, residuals auto-correlation function (ACF) plot, and residuals histogram for adjusted ARIMAX(0,1,1) model for the outcome of mean daily FM cigarette consumption as the outcome variable.

- Ljung-Box test results for residuals from ARIMAX(0,1,1) for the outcome of mean daily FM cigarette consumption with non-zero mean: Q*=25.19, df=23, p=0.341 → no significant autocorrelation.

Table S6: Best fitting model for mean daily FM cigarette consumption as the outcome variable – ARIMAX(0,1,1).

Variables	Unadjusted model		Adjusted model	
	B (95% CI)	p-value	B (95% CI)	p-value
MA1	-0.861 (-0.928, -0.793)	<0.001	-0.875 (-0.944, -0.805)	<0.001
Standardised packaging (intervention effect modelled using a gradual level change)	-0.681 (-1.584, 0.222)	0.139	-0.543 (-1.381, 0.296)	0.204
Tobacco control policies (comprised score as dummy variable)	NA	NA	-0.357 (-1.112, 0.398)	0.354
Tax increases (transfer function, lag 3)	NA	NA	0.318 (-0.214, 0.851)	0.241
Mass media expenditure (transfer function, lag 0)	NA	NA	-0.134 (-0.487, 0.218)	0.455

The Bayes factor for the adjusted Beta coefficient for the standardised packaging was 1.02, which suggests that the evidence about the impact of the standardised packaging implementation on mean daily FM cigarette consumption was inconclusive.

Sensitivity analysis 1a: ARIMAX(0,1,1) with lag 0 for all covariates

Sensitivity analysis with the adjusted ARIMAX(0,1,1) model including a lag of 0 for all covariates as cross correlation function plots did not show any strong correlation between mean daily FM cigarette consumption and the respective covariates.

Table S7: Results for sensitivity analysis with lag 0 for all covariates, with mean daily FM cigarette consumption as the outcome variable.

Variables	Adjusted model	
	B (95% CI)	p-value
MA1	-0.884 (-0.955, -0.813)	<0.001
Standardised packaging (intervention effect modelled using a gradual level change)	-0.450 (-1.351, 0.450)	0.327
Tobacco control policies (comprised score as dummy variable)	-0.402 (-1.167, 0.363)	0.303
Tax increases (transfer function, lag 0)	-4.413 (-11.316, 2.490)	0.210
Mass media expenditure (transfer function, lag 0)	-0.153 (-0.511, 0.205)	0.403

The Box-Ljung test indicates no significant autocorrelation for the model **(**Q*=22.24, df=23, p=0.506).

Sensitivity analysis 1b: ARIMAX(0,0,1)

Although the Augmented Dickey-Fuller test indicated that no differencing is required, the time series plot (see Figure S1) indicated that the series is non-stationary. Therefore, the main analysis included 1-lag difference. As a sensitivity, test without differencing – ARIMAX(0,0,1).

Table S8: Results for sensitivity analysis with model ARIMAX(0,0,1) and covariates the same as in the main analysis, with mean daily FM cigarette consumption as the outcome variable.

Variables	Unadjusted model		Adjusted model	
	B (95% CI)	p-value	B (95% CI)	p-value
MA1	0.283 (0.165, 0.402)	<0.001	-0.073 (-0.224, 0.077)	0.338
Intercept term	11.504 (11.298, 11.710)	<0.001	13.542 (13.081, 14.004)	<0.001
Standardised packaging (intervention effect modelled using a gradual level change)	-1.437 (-1.939, -0.934)	<0.001	-0.276 (-0.644, 0.092)	0.141
Tobacco control policies (comprised score as dummy variable)	NA	NA	-0.516 (-1.268, 0.236)	0.179
Tax increases (transfer function, lag 3)	NA	NA	0.986 (0.956, 1.016)	<0.001
Mass media expenditure (transfer function, lag 0)	NA	NA	-0.206 (-0.512, 0.099)	0.185

The Box-Ljung test indicates significant autocorrelation for the unadjusted model **(**Q*=295.5, df=23, p<0.001) and the ACF plot of the residuals shows several significant lags. Hence, the model does not fit well.

Sensitivity analysis 1c: alternative gradual level shift

Pre-planned sensitivity analysis with alternative gradual level shift that starts in June 2016 until May 2017 (i.e., from November 2007 until May 2016 as 0, from June 2016 until May 2017 each month increase by 1/12 till June 2017, from then onwards as 1).

Table S9: Results for sensitivity analysis using alternative gradual level change with model ARIMAX(0,1,1) and covariates the same as in the main analysis, with mean daily FM cigarette consumption as the outcome variable.

Variables	Unadjusted model		Adjusted model	
	B (95% CI)	p-value	B (95% CI)	p-value
MA1	-0.854 (-0.926, -0.782)	<0.001	-1.000 (-1.038, -0.962)	<0.001
Standardised packaging (intervention effect modelled using a gradual level change)	-0.374 (-1.312, 0.563)	0.434	-0.071 (-0.515, 0.373)	0.753
Tobacco control policies (comprised score as dummy variable)	NA	NA	-0.465 (-1.217, 0.288)	0.227
Tax increases (transfer function, lag 3)	NA	NA	0.989 (0.953, 1.026)	<0.001
Mass media expenditure (transfer function, lag 0)	NA	NA	-0.163 (-0.494, 0.167)	0.333

The Box-Ljung test indicates no significant autocorrelation for the time-series in the unadjusted **(**Q*=22.88, df=23, p=0.468) and adjusted model **(**Q*=21.13, df=23, p=0.573).

Sensitivity analysis 1d: alternative step change

Pre-planned sensitivity analysis with alternative step change in June 2017 (i.e., from November 2007 until May 2017 as 0, and from June 2017 onwards as 1).

Table S10: Results for sensitivity analysis using alternative step change with model ARIMAX(0,1,1) and covariates the same as in the main analysis, with mean daily FM cigarette consumption as the outcome variable.

Variables	Unadjusted model		Adjusted model	
	B (95% CI)	p-value	B (95% CI)	p-value
MA1	-0.854 (-0.928, -0.779)	<0.001	-1.000 (-1.037, -0.962)	<0.001
Standardised packaging (intervention effect modelled using a step change)	-0.134 (-0.883, 0.615)	0.725	-0.120 (-0.504, 0.264)	0.541
Tobacco control policies (comprised score as dummy variable)	NA	NA	-0.461 (-1.213, 0.292)	0.230
Tax increases (transfer function, lag 3)	NA	NA	0.988 (0.951, 1.024)	<0.001
Mass media expenditure (transfer function, lag 0)	NA	NA	-0.169 (-0.495, 0.158)	0.312

The Box-Ljung test indicates no significant autocorrelation for the time-series in the unadjusted (Q*=24.05, df=23, p=0.401) and adjusted model **(**Q*=21.77, df=23, p=0.534).

Sensitivity analysis 1e: exclusive FM cigarette consumption

Pre-planned sensitivity analysis restricting the outcome to only those who exclusively smoked FM cigarettes rather than predominantly.

Table S11: Results for sensitivity analysis for exclusive FM cigarette consumption as the outcome variable with model ARIMAX(0,1,1) and covariates the same as in the main analysis

Variables	Unadjusted model		Adjusted model	
	B (95% CI)	p-value	B (95% CI)	p-value
MA1	-0.853 (-0.925, -0.782)	<0.001	-0.999 (-1.042, -0.956)	<0.001
Standardised packaging (intervention effect modelled using a gradual level change)	-0.657 (-1.624, 0.310)	0.183	-0.221 (-0.630, 0.188)	0.290
Tobacco control policies (comprised score as dummy variable)	NA	NA	-0.543 (-1.317, 0.231)	0.169
Tax increases (transfer function, lag 3)	NA	NA	0.987 (0.955, 1.018)	<0.001
Mass media expenditure (transfer function, lag 0)	NA	NA	-0.180 (-0.516, 0.155)	0.292

The Box-Ljung test indicates no significant autocorrelation for the time-series in the unadjusted (Q*=23.85, df=23, p=0.412) and adjusted model (Q*=22.68, df=23, p=0.480).

Sensitivity analysis 1f: FM cigarette consumption among all who smoke cigarettes

Sensitivity analysis with the outcome being mean daily FM cigarette consumption among all who smoke cigarettes.

Table S12: Results for sensitivity analysis for FM cigarette consumption among all who smoke cigarettes as the outcome variable with model ARIMAX(0,1,1) and covariates the same as in the main analysis

Variables	Unadjusted model		Adjusted model	
	B (95% CI)	p-value	B (95% CI)	p-value
MA1	-0.781 (-0.862, -0.699)	<0.001	-0.997 (-1.046, -0.947)	<0.001
Standardised packaging (intervention effect modelled using a gradual level change)	-0.524 (-1.554, 0.505)	0.318	0.021 (-0.317, 0.359)	0.903
Tobacco control policies (comprised score as dummy variable)	NA	NA	0.065 (-0.521, 0.650)	0.828
Tax increases (transfer function, lag 3)	NA	NA	0.999 (0.984, 1.014)	<0.001
Mass media expenditure (transfer function, lag 0)	NA	NA	-0.205 (-0.458, 0.047)	0.111

The Box-Ljung test indicates no significant autocorrelation for the time-series in the unadjusted (Q*=31.88, df=23, p=0.103) and adjusted model (Q*=20.84, df=23, p=0.591).

Results for mean daily roll-your-own (RYO) cigarette consumption

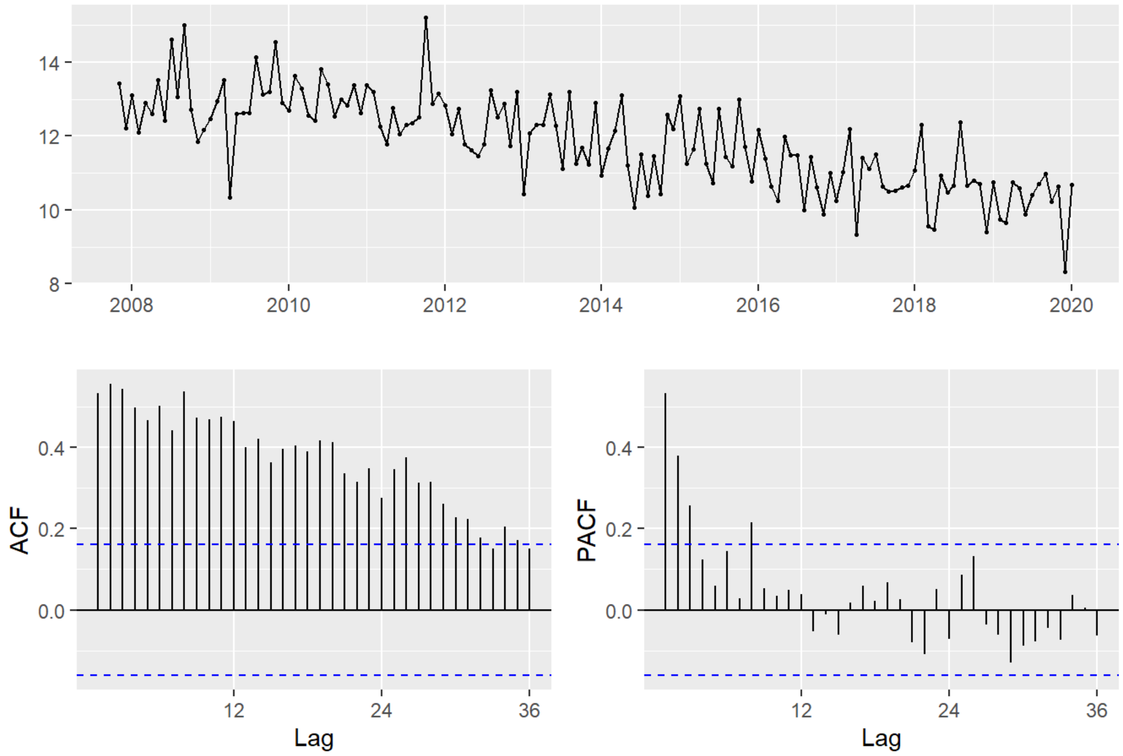

Figure S5: Time series, auto-correlation function (ACF), and partial auto-correlation function (PACF) graphs of mean daily RYO cigarette consumption as the outcome variable.

- Augmented Dickey-Fuller tests shows no differences required to make series stationary.
- As time series in Figure S5 indicates that differencing might be required, use lag-1 difference in main analysis with further sensitivity analysis without lag-1 difference.
- Seasonal unit root tests show no seasonal differences required to make series seasonally stationary.

Table S13: Akaike information criterion (AIC) for different ARIMAX models for the outcome of mean daily RYO cigarette consumption (unadjusted).

ARIMAX	AIC
(1,1,0)	417.7
(0,1,1)	**378.4**
(1,1,1)	380.0
(2,1,0)	401.1
(0,1,2)	380.0
(2,1,1)	381.7
(1,1,2)	381.1

➔ Smallest AIC for ARIMAX(0,1,1); choose this model

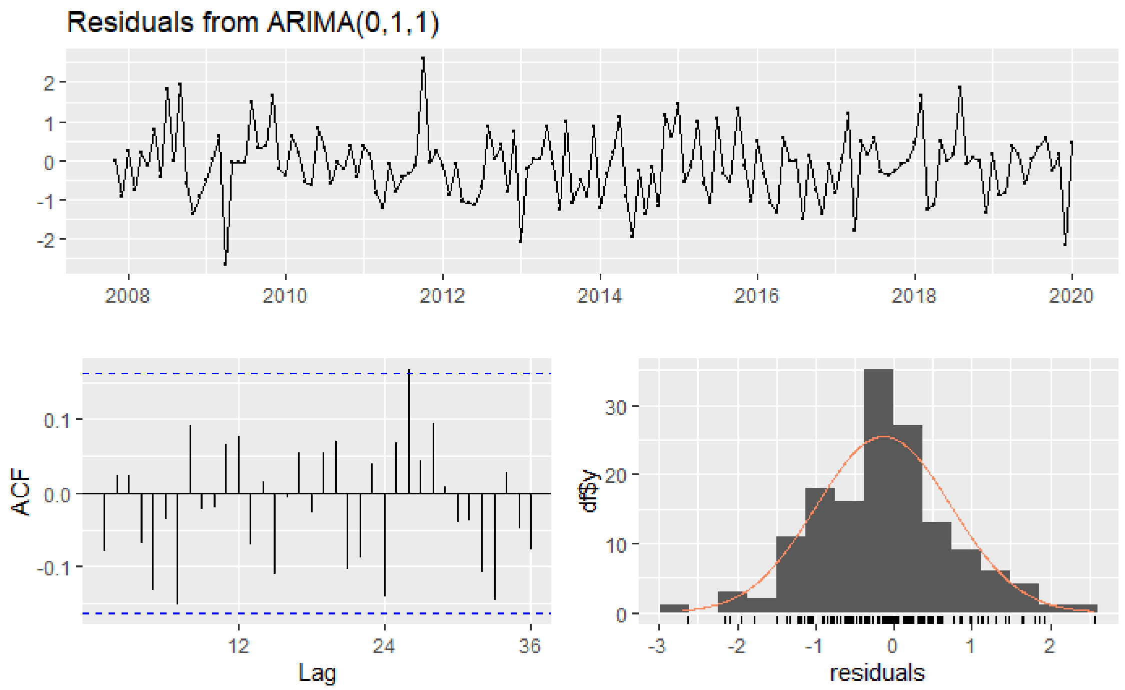

Figure S6: Residuals time series plot, residuals auto-correlation function (ACF) plot, and residuals histogram for unadjusted ARIMAX(0,1,1) model for mean RYO cigarette consumption as the outcome variable.

Ljung-Box test results for residuals from unadjusted ARIMAX(0,1,1) for mean daily RYO cigarette consumption as the outcome variable with non-zero mean: Q*=23.17, df=23, p=0.451 → no significant autocorrelation.

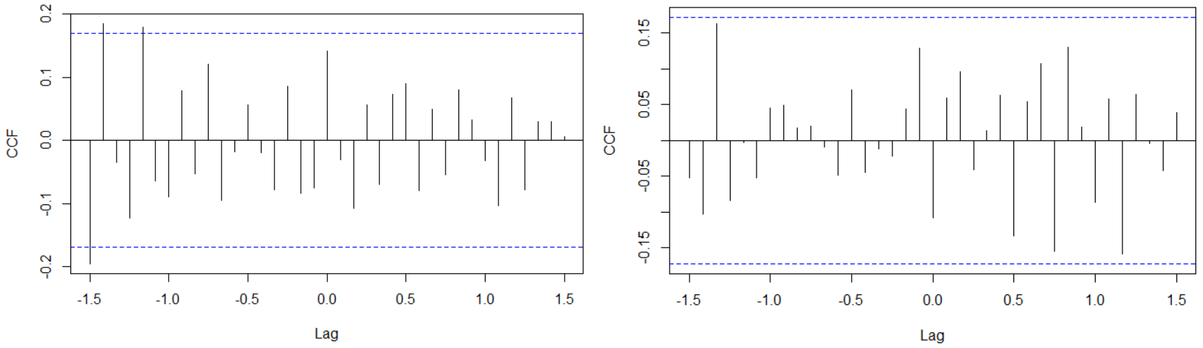

Figure S7: Cross correlation function plots for mean daily RYO cigarette consumption time series and (1) tax increase time series, and (2) mass media expenditure time series (from left to right).

Table S14: AIC values for mass media expenditure included as transfer functions in the ARIMAX(0,1,1) model for mean daily RYO cigarette consumption as the outcome variable using different numbers of AR terms.

AR terms	Tax increases, AIC	Mass media expenditure, AIC
0	**366.3**	381.9
1	367.9	382.4
2	369.0	378.7
3	371.0	**377.7**

- For tax increases, smallest AIC with 0 lags
- For mass media expenditure, smallest AIC with 3 lags, but less than 2 units difference to 2 lags. Hence, test whether 2 lags still have the lowest AIC when lag 1 is fixed to zero → not the smallest AIC anymore (AIC=383.1) → go with 0 lags.

➔ Adjusted model: ARIMAX(0,1,1) with dummy variable for tobacco control policies, transfer function for tax increases (0,0), and transfer function for mass media expenditure (0,0).

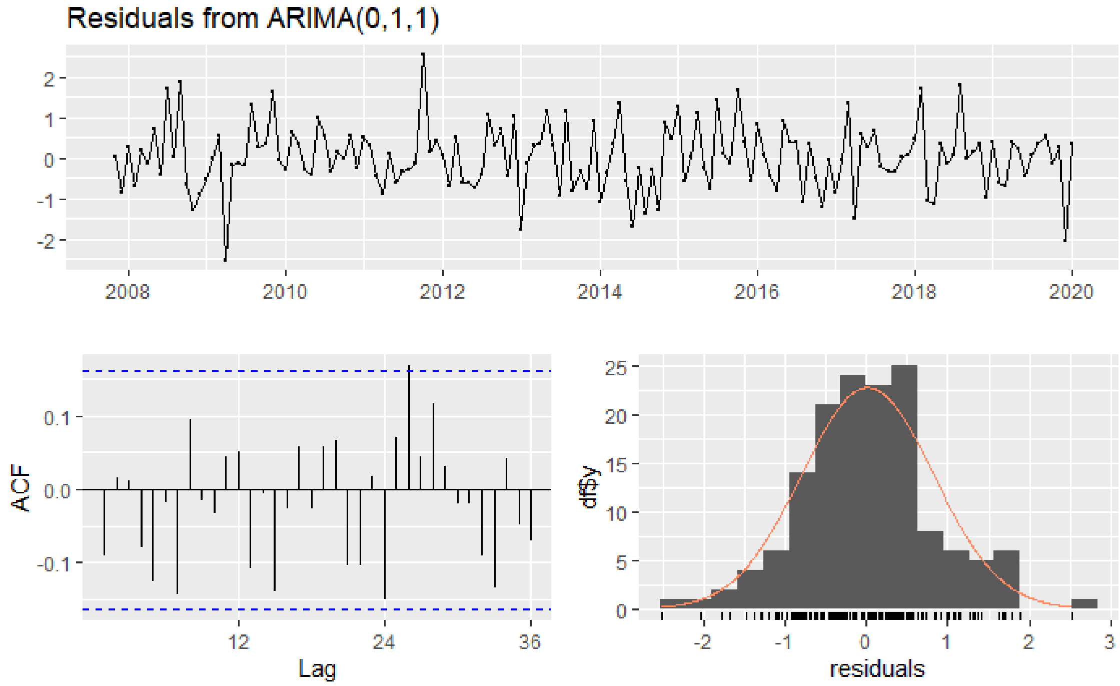

Figure S8: Residuals time series plot, residuals auto-correlation function (ACF) plot, and residuals histogram for adjusted ARIMAX(0,1,1) model for the outcome of mean RYO cigarette consumption.

Ljung-Box test results for residuals from adjusted ARIMAX(0,1,1) for mean RYO cigarette consumption as the outcome variable with non-zero mean: Q*=25.34, df=23, p=0.333 → no significant autocorrelation.

Table S15: Best fitting model for the outcome of mean daily RYO cigarette consumption – ARIMAX(0,1,1).

Variables	Unadjusted model		Adjusted model	
	B (95% CI)	p-value	B (95% CI)	p-value
MA1	-0.886 (-0.951, -0.821)	<0.001	-1.000 (-1.035, -0.965)	<0.001
Standardised packaging (intervention effect modelled using a gradual level change)	-0.473 (-1.472, 0.527)	0.354	0.002 (-0.518, 0.522)	0.994
Tobacco control policies (comprised score as dummy variable)	NA	NA	0.143 (-0.794, 1.079)	0.765
Tax increases (transfer function, lag 0)	NA	NA	-9.725 (-11.713, -7.737)	<0.001
Mass media expenditure (transfer function, lag 0)	NA	NA	0.085 (-0.320, 0.490)	0.682

The Bayes factor for the adjusted coefficient for the standardised packaging was 0.80, suggesting that the evidence of whether the introduction of standardised packaging had an impact on mean daily RYO cigarette consumption is inconclusive

Sensitivity analysis 2a: ARIMAX(0,0,1)

Although the Augmented Dickey-Fuller test indicated that no differencing is required, the time series plot (see Figure S5) indicated that the series is non-stationary. Therefore, the main analysis included 1-lag difference. As a sensitivity, test without differencing – ARIMAX(0,0,1).

Table S16: Results for sensitivity analysis with model ARIMAX(0,0,1), with mean daily RYO cigarette consumption as the outcome variable.

Variables	Unadjusted model		Adjusted model	
	B (95% CI)	p-value	B (95% CI)	p-value
MA1	0.229 (0.097, 0.360)	0.001	-0.092 (-0.251, 0.067)	0.257
Intercept term	12.180 (11.959, 12.401)	<0.001	22.677 (20.697, 24.658)	<0.001
Standardised packaging (intervention effect modelled using a gradual level change)	-1.838 (-2.378, -1.298)	<0.001	0.007 (-0.462, 0.477)	0.975
Tobacco control policies (comprised score as dummy variable)	NA	NA	0.144 (-0.787, 1.075)	0.761
Tax increases (transfer function, lag 0)	NA	NA	-9.735 (-11.530, -7.940)	<0.001
Mass media expenditure (transfer function, lag 0)	NA	NA	0.095 (-0.283, 0.473)	0.622

The Box-Ljung test indicates significant autocorrelation for the unadjusted model **(**Q*=145.72, df=23, p<0.001) and the ACF plot of the residuals shows several significant lags. Hence, the model does not fit well.

Sensitivity analysis 2b: alternative gradual level shift

Pre-planned sensitivity analysis with alternative gradual level shift that starts in June 2016 until May 2017 (i.e., from November 2007 until May 2016 as 0, from June 2016 until May 2017 each month increase by 1/12 till June 2017, from then onwards as 1).

Table S17: Results for sensitivity analysis using alternative gradual level change with model ARIMAX(0,1,1) and covariates the same as in the main analysis, with mean daily RYO cigarette consumption as the outcome variable.

Variables	Unadjusted model		Adjusted model	
	B (95% CI)	p-value	B (95% CI)	p-value
MA1	-0.896 (-0.959, -0.834)	<0.001	-1.000 (-1.035, -0.965)	<0.001
Standardised packaging (intervention effect modelled using a gradual level change)	-0.713 (-1.657, 0.230)	0.139	-0.065 (-0.613, 0.483)	0.815
Tobacco control policies (comprised score as dummy variable)	NA	NA	0.138 (-0.799, 1.075)	0.773
Tax increases (transfer function, lag 0)	NA	NA	-9.499 (-11.871, -7.127)	<0.001
Mass media expenditure (transfer function, lag 0)	NA	NA	0.073 (-0.336, 0.483)	0.725

The Box-Ljung test indicates no significant autocorrelation for the time-series in the unadjusted (Q*=22.69, df=23, p =0.479) and adjusted model (Q*=25.26, df=23, p =0.337).

Sensitivity analysis 2c: alternative step change

Pre-planned sensitivity analysis with alternative step change in June 2017 (i.e., from November 2007 until May 2017 as 0, and from June 2017 onwards as 1).

Table S18: Results for sensitivity analysis using alternative step change with model ARIMAX(0,1,1) and covariates the same as in the main analysis, with mean daily RYO cigarette consumption as the outcome variable.

Variables	Unadjusted model		Adjusted model	
	B (95% CI)	p-value	B (95% CI)	p-value
MA1	-0.881 (-0.949, -0.814)	<0.001	-1.000 (-1.035, -0.965)	<0.001
Standardised packaging (intervention effect modelled using a step change)	-0.195 (-1.048, 0.658)	0.655	0.055 (-0.424, 0.535)	0.821
Tobacco control policies (comprised score as dummy variable)	NA	NA	0.145 (-0.791, 1.082)	0.761
Tax increases (transfer function, lag 0)	NA	NA	-9.886 (-11.942, -7.830)	<0.001
Mass media expenditure (transfer function, lag 0)	NA	NA	0.093 (-0.313, 0.498)	0.654

The Box-Ljung test indicates no significant autocorrelation for the time-series in the unadjusted (Q*=23.47, df=23, p=0.434) and adjusted model (Q*=25.41, df=23, p=0.330).

Sensitivity analysis 2d: exclusive RYO cigarette consumption

Pre-planned sensitivity analysis restricting the outcome to only those who exclusively smoked RYO cigarettes rather than predominantly.

Table S19: Results for sensitivity analysis for exclusive RYO cigarette consumption as the outcome variable with model ARIMAX(0,1,1) and covariates the same as in the main analysis.

Variables	Unadjusted model		Adjusted model	
	B (95% CI)	p-value	B (95% CI)	p-value
MA1	-0.879 (-0.945, -0.813)	<0.001	-1.000 (-1.035, -0.965)	<0.001
Standardised packaging (intervention effect modelled using gradual level change)	-0.498 (-1.517, 0.575)	0.363	0.073 (-0.466, 0.612)	0.792
Tobacco control policies (comprised score as dummy variable)	NA	NA	0.085 (-0.887, 1.056)	0.864
Tax increases (transfer function, lag 0)	NA	NA	-11.021 (-13.083, -8.959)	<0.001
Mass media expenditure (transfer function, lag 0)	NA	NA	0.129 (-0.291, 0.549)	0.548

The Box-Ljung test indicates significant autocorrelation for the time-series in the unadjusted (Q*=25.86, df=23, p=0.308) and adjusted model (Q*=27.12, df=23, p=0.251).

Sensitivity analysis 2e: RYO cigarette consumption among all who smoke cigarettes

Sensitivity analysis with the outcome mean daily RYO cigarette consumption among all who smoke cigarettes.

Table S20: Results for sensitivity analysis for the outcome of RYO cigarette consumption among all who smoke with model ARIMAX(0,1,1) and covariates the same as in the main analysis.

Variables	Unadjusted model		Adjusted model	
	B (95% CI)	p-value	B (95% CI)	p-value
MA1	-0.861 (-0.940, -0.782)	<0.001	-0.886 (-0.971, -0.802)	<0.001
Standardised packaging (intervention effect modelled using a gradual level change)	-0.027 (-0.675, 0.622)	0.936	-0.345 (-0.894, 0.205)	0.219
Tobacco control policies (comprised score as dummy variable)	NA	NA	-0.187 (-0.835, 0.461)	0.572
Tax increases (transfer function, lag 0)	NA	NA	2.826 (-1.864, 7.517)	0.238
Mass media expenditure (transfer function, lag 0)	NA	NA	0.150 (-0.106, 0.406)	0.249

The Box-Ljung test indicates significant autocorrelation for the time-series in the unadjusted ($Q^*=23.79$, df=23, p=0.416) and adjusted model ($Q^*=22.57$, df=23, p=0.486).

Results for current smoking among 16-to-24-year-olds

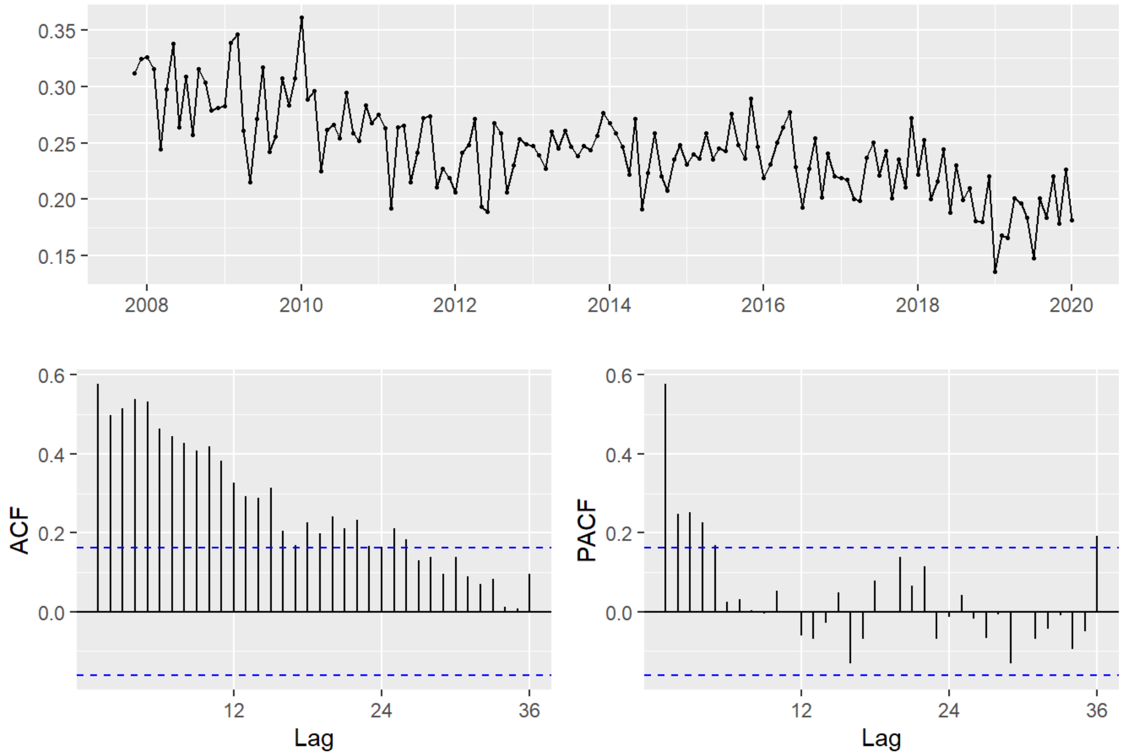

Figure S9: Time series, auto-correlation function (ACF), and partial auto-correlation function (PACF) graphs of current smoking among 16-to-24-year-olds as the outcome variable.

- Augmented Dickey-Fuller tests shows no differences required to make series stationary.
- As time series in Figure S9 indicates that differencing might be required, main analysis with lag-1 difference, and further sensitivity analysis without lag-1 difference.
- Seasonal unit root tests show no seasonal differences required to make series seasonally stationary.

Table S21: Akaike information criterion (AIC) for different ARIMAX models for current smoking among 16-to-24-year-olds as the outcome variable (unadjusted).

ARIMAX	AIC
(1,1,0)	-581.0
(0,1,1)	**-624.7**
(1,1,1)	-622.8
(2,1,0)	-600.7
(0,1,2)	-622.8
(2,1,1)	-623.6
(1,1,2)	-621.8

➔ Smallest AIC for ARIMAX(0,1,1)

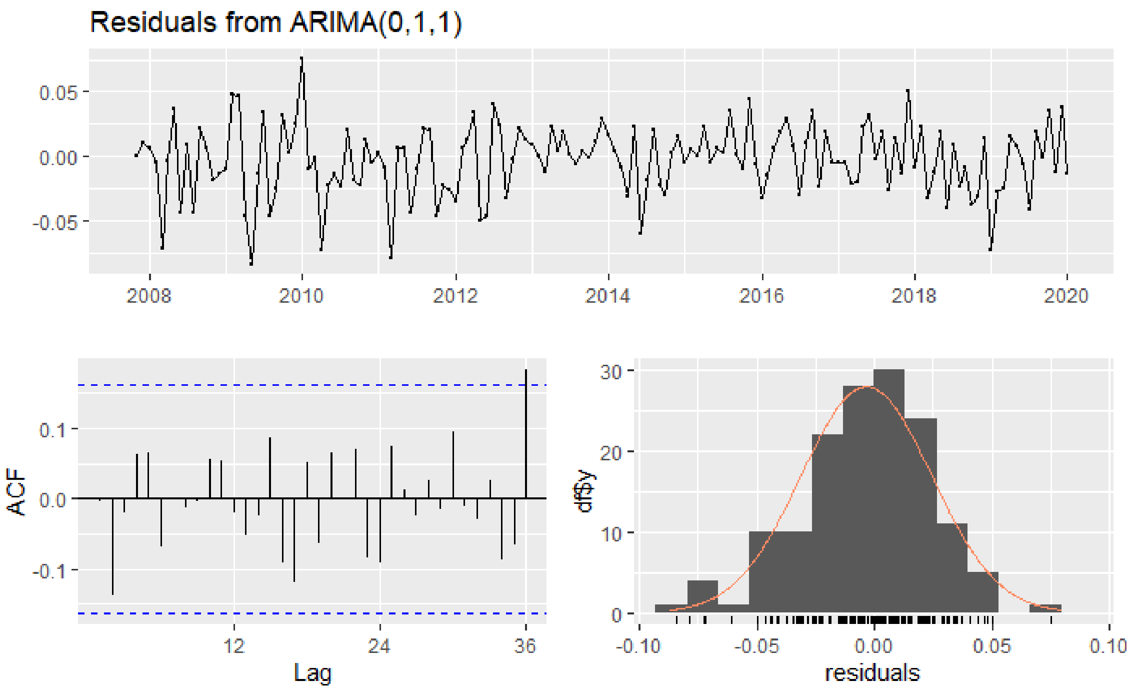

Figure S10: Residuals time series plot, residuals auto-correlation function (ACF) plot, and residuals histogram for unadjusted ARIMAX(0,1,1) model for current smoking among 16-to-24-year-olds as the outcome variable.

Ljung-Box test results for residuals from unadjusted ARIMAX(0,1,1) for current smoking among 16-to-24-year-olds as the outcome variable with non-zero mean: Q*=17.11, df=23, p=0.804 → no significant autocorrelation.

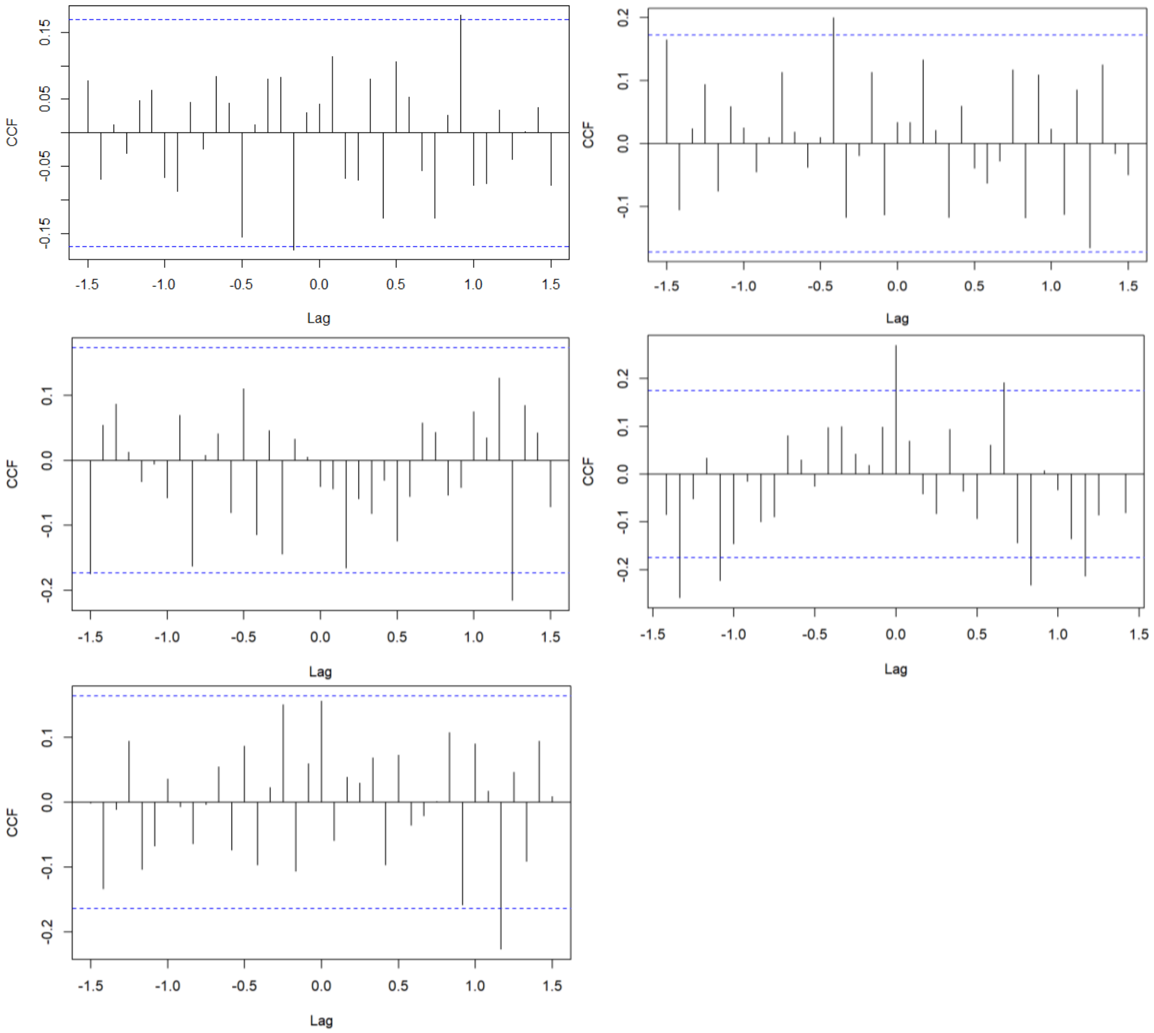

Figure S11: Cross correlation function plot for the current smoking among 16-to-24-year-olds time series and (1) tax increase time series, (2) mass media expenditure time series, (3) use of prescription medication in quit attempt, (4) use of over-the-counter medication or behavioural support in quit attempt, (5) use of e-cigarettes (from top left to bottom right).

Table S22: AIC values for covariates included as transfer functions in the ARIMAX model for current smoking among 16-to-24-year-olds as the outcome variable using different numbers of AR terms.

AR terms	Tax increases, AIC	Mass media expenditure, AIC	Use of prescription medication in quit attempt, AIC	Use of over-the-counter medication/ behavioural support in quit attempt, AIC	E-cigarette use, AIC
0	-623.6	-625.4	-622.8	**-632.4**	**-627.8**
1	-624.5	-624.3	-620.8	-630.4	-625.9
2	-621.4	-625.0	**-630.1**	-628.6	-625.6
3	**-626.5**	**-628.8**	-628.1	-629.9	-625.3

- For tax increases, lowest AIC for 3 lags. When 1st and 2nd lag fixed to zero, no longer smallest (AIC=-624.0) → go with 0 lags.
- For mass media expenditure, lowest AIC for 3 lags. When 1st and 2nd lag are fixed to zero, no longer smallest (AIC=-623.4) → go with 0 lags.
- For use of prescription medication in quit attempt, lowest AIC for 2 lags. When 1st lag is fixed to zero, no longer smallest (AIC=-621.7) → go with 0 lags.

- For use of over-the-counter medication or behavioural support in quit attempt and e-cigarette use, lowest AICs for 0 lags.
- Adjusted model: ARIMAX(0,1,1) with dummy variable for tobacco control policies, and transfer functions for tax increases (0,0), mass media expenditure (0,0), use of prescription medication in quit attempt (0,0), use of over-the-counter medication/behavioural support in quit attempt (0,0), and e-cigarette use (0,0)

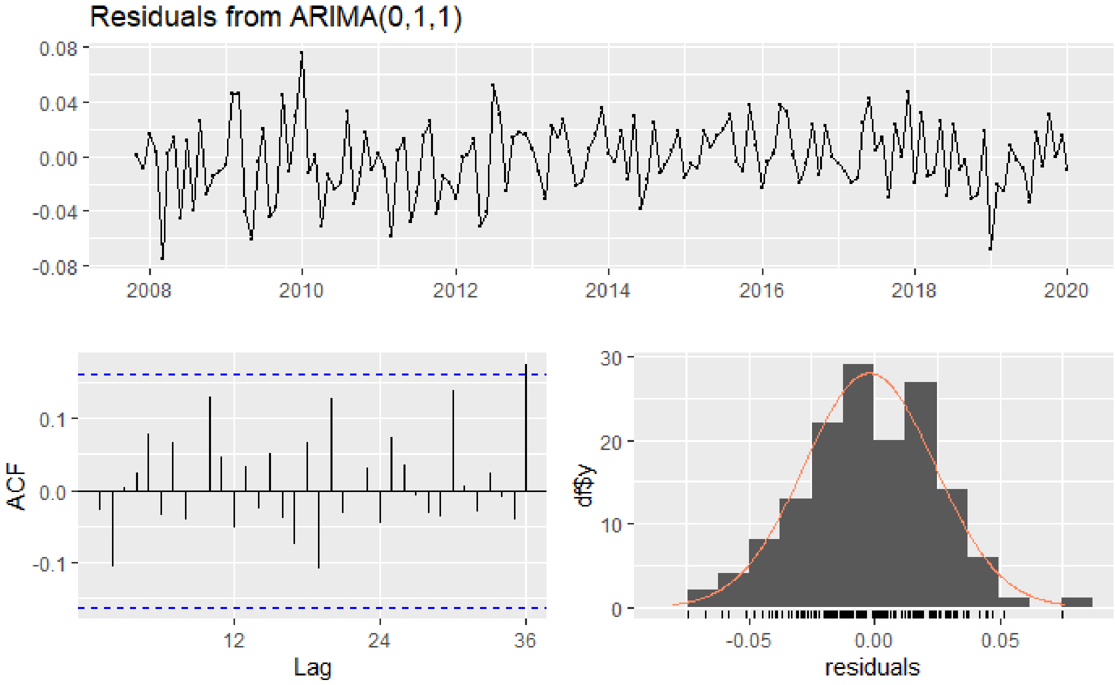

Figure S12: Residuals time series plot, residuals auto-correlation function (ACF) plot, and residuals histogram for adjusted ARIMAX(0,1,1) model for current smoking among 16-to-24-year-olds as the outcome variable.

Ljung-Box test results for residuals from adjusted ARIMAX(0,1,1) for current smoking among 16-to-24-year-olds as the outcome variable with non-zero mean: Q*=15.63, df=23, p=0.871 → no significant autocorrelation.

Table S23: Best fitting model for the outcome of current smoking among 16-to-24-year-olds – ARIMAX(0,1,1).

Variables	Unadjusted model		Adjusted model	
	B (95% CI)	p-value	B (95% CI)	p-value
MA1	-0.828 (-0.914, -0.741)	<0.001	-0.877 (-0.958, -0.797)	<0.001
Standardised packaging (intervention effect modelled using a step change)	-0.031 (-0.062, 0.000)	0.047	-0.010 (-0.039, 0.019)	0.497
Tobacco control policies (comprised score as dummy variable)	NA	NA	0.020 (-0.009, 0.050)	0.170
Tax increases (transfer function, lag 0)	NA	NA	-0.242 (-0.502, 0.019)	0.069
Mass media expenditure (transfer function, lag 0)	NA	NA	0.007 (-0.007, 0.021)	0.318
Use of prescription medication in quit attempt (transfer function, lag 0)	NA	NA	-0.054 (-0.282, 0.173)	0.640

Variables	Unadjusted model		Adjusted model	
	B (95% CI)	p-value	B (95% CI)	p-value
Use of over-the-counter medication or behavioural support in quit attempt (transfer function, lag 0)	NA	NA	-0.583 (0.214, 0.953)	0.002
E-cigarette use (transfer function, lag 0)	NA	NA	0.480 (0.116, 0.884)	0.010

Sensitivity analysis 3a: ARIMAX(0,0,1)

Although the Augmented Dickey-Fuller test indicated that no differencing is required, the time series plot (see Figure S9) indicated that the series is non-stationary. Therefore, the model in the main analysis is with 1-lag difference, and the sensitivity test without differencing – ARIMAX(0,0,1).

Table S24: Results for sensitivity analysis with model ARIMAX(0,0,1) and covariates the same as in the main analysis, with current smoking among 16-to-24-year-olds as the outcome variable.

Variables	Unadjusted model		Adjusted model	
	B (95% CI)	p-value	B (95% CI)	p-value
MA1	0.318 (0.179, 0.457)	<0.001	0.109 (-0.070, 0.287)	0.234
Intercept term	0.259 (0.251, 0.267)	<0.001	0.573 (0.402, 0.743)	<0.001
Standardised packaging (intervention effect modelled using a step change)	-0.050 (-0.065, -0.036)	<0.001	0.012 (-0.010, 0.034)	0.267
Tobacco control policies (comprised score as dummy variable)	NA	NA	0.021 (-0.009, 0.052)	0.173
Tax increases (transfer function, lag 0)	NA	NA	-0.329 (-0.487, -0.172)	<0.001
Mass media expenditure (transfer function, lag 0)	NA	NA	0.009 (-0.006, 0.024)	0.224
Use of prescription medication in quit attempt (transfer function, lag 0)	NA	NA	-0.077 (-0.321, 0.167)	0.535
Use of over-the-counter medication or behavioural support in quit attempt (transfer function, lag 0)	NA	NA	0.700 (0.302, 1.097)	0.001
E-cigarette use (transfer function, lag 0)	NA	NA	0.555 (0.215, 0.896)	0.001

The Box-Ljung test indicates significant autocorrelation for the unadjusted model ($Q^*=82.11$, $df=23$, $p<0.001$) and the ACF plot of the residuals shows several significant lags. Hence, the model does not fit well.

Sensitivity analysis 3b: alternative step change

Pre-planned sensitivity analysis with alternative step change in June 2017 (i.e., from November 2007 until May 2017 as 0, and from June 2017 onwards as 1).

Table S25: Results for sensitivity analysis using alternative step change with model ARIMAX(0,1,1) and covariates the same as in the main analysis, with current smoking among 16-to-24-year-olds as the outcome variable.

Variables	Unadjusted model		Adjusted model	
	B (95% CI)	p-value	B (95% CI)	p-value
MA1	-0.814 (-0.903, -0.724)	<0.001	-0.884 (-0.959, -0.808)	<0.001
Standardised packaging (intervention effect modelled using step change)	0.008 (-0.026, 0.042)	0.644	0.013 (-0.013, 0.039)	0.334
Tobacco control policies (comprised score as dummy variable)	NA	NA	0.021 (-0.008, 0.051)	0.150
Tax increases (transfer function, lag 0)	NA	NA	-0.323 (-0.568, -0.078)	0.010
Mass media expenditure (transfer function, lag 0)	NA	NA	0.007 (-0.006, 0.021)	0.293
Use of prescription medication in quit attempt (transfer function, lag 0)	NA	NA	-0.056 (-0.284, 0.172)	0.631
Use of over-the-counter medication or behaviour support in quit attempt (transfer function, lag 0)	NA	NA	0.594 (0.228, 0.960)	0.001
E-cigarette use (transfer function, lag 0)	NA	NA	0.538 (0.186, 0.890)	0.003

The Ljung-Box test indicates no significant autocorrelation for the time-series in the unadjusted (Q*=17.52, df=23, p =0.783) and adjusted model (Q*=15.71, df=23, p =0.868).

Sensitivity analysis 3c: alternative gradual level change

Pre-planned sensitivity analysis with alternative gradual level shift that starts in June 2016 until May 2017 (i.e., from November 2007 until May 2016 as 0, from June 2016 until May 2017 each month increase by 1/12 till June 2017, from then onwards as 1).

Table S26: Results for sensitivity analysis using alternative gradual level change with model ARIMAX(0,1,1) and covariates the same as in the main analysis, with current smoking among 16-to-24-year-olds as the outcome variable.

Variables	Unadjusted model		Adjusted model	
	B (95% CI)	p-value	B (95% CI)	p-value
MA1	-0.830 (-0.918, -0.741)	<0.001	-0.887 (-0.963, -0.812)	<0.001
Standardised packaging (intervention effect, modelled using a gradual level change)	-0.023 (-0.065, 0.019)	0.284	0.002 (-0.032, 0.036)	0.912
Tobacco control policies (comprised score as dummy variable)	NA	NA	0.021 (-0.008, 0.050)	0.161
Tax increases (transfer function, lag 0)	NA	NA	-0.287 (-0.546, 0.028)	0.030

Variables	Unadjusted model		Adjusted model	
	B (95% CI)	p-value	B (95% CI)	p-value
Mass media expenditure (transfer function, lag 0)	NA	NA	0.007 (-0.006, 0.021)	0.297
Use of prescription medication in quit attempt (transfer function, lag 0)	NA	NA	-0.060 (-0.289, 0.169)	0.609
Use of over-the-counter medication or behaviour support in quit attempt (transfer function, lag 0)	NA	NA	0.594 (0.225, 0.964)	0.002
E-cigarette use (transfer function, lag 0)	NA	NA	0.527 (0.165, 0.888)	0.004

The Ljung-Box test indicates no significant autocorrelation for the time-series in the unadjusted (Q*=17.18, df=23, p=0.800) and adjusted model (Q*=15.12, df=23, p=0.890).

Sensitivity analysis 3d: current smoking among those aged 25 years and older

Pre-planned sensitivity analysis running the model from the main analysis for 16-to-24-year-olds using a sample of people aged 25 years and over as a comparison to test whether observed effect specific to young people or all age groups.

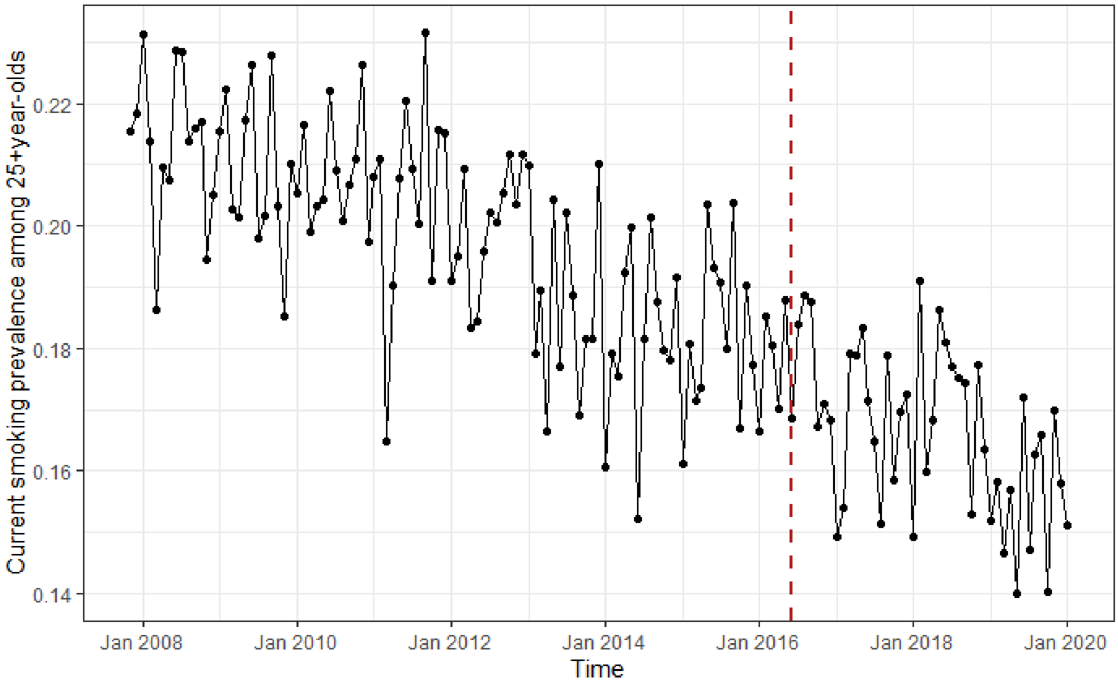

Figure S13: Time-series for the outcome of current smoking prevalence among people aged 25 years and over. The red line indicates the month when the policy (standardised packaging) was introduced.

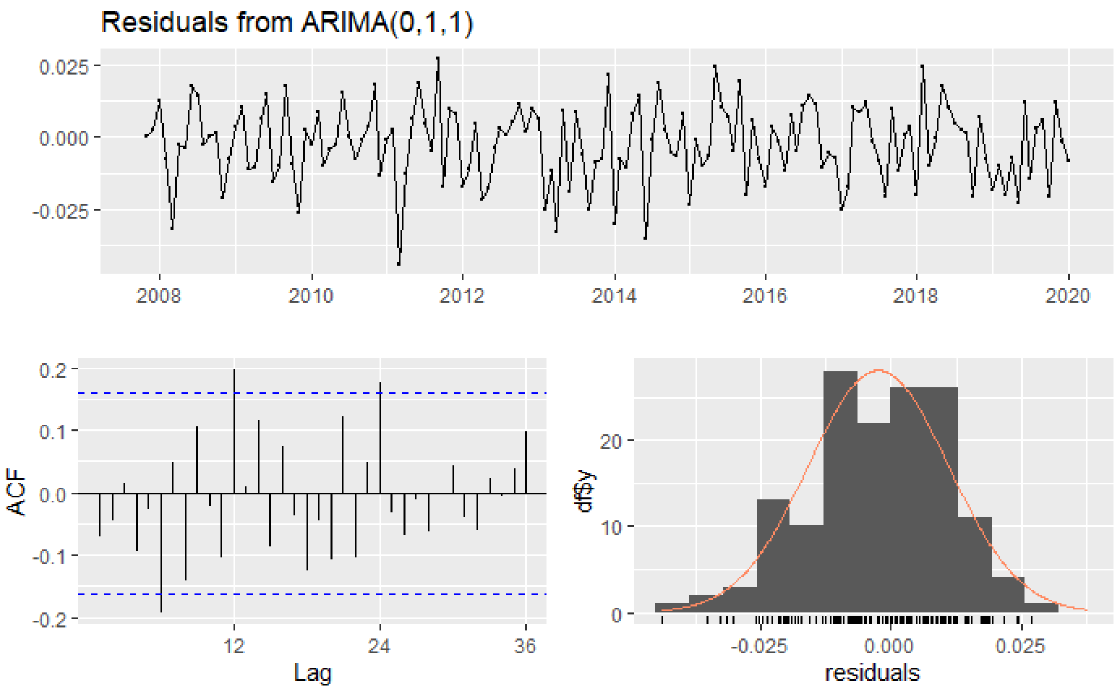

Figure S14: Residuals time series plot, residuals auto-correlation function (ACF) plot, and residuals histogram for unadjusted ARIMAX(0,1,1) model for the outcome of current smoking among those aged 25+ years.

Ljung-Box test results for residuals from unadjusted ARIMAX(0,1,1) for current smoking among adults aged 25+ years as the outcome variable with non-zero mean: Q*=41.70, df=23, p=0.010 → significant autocorrelation.

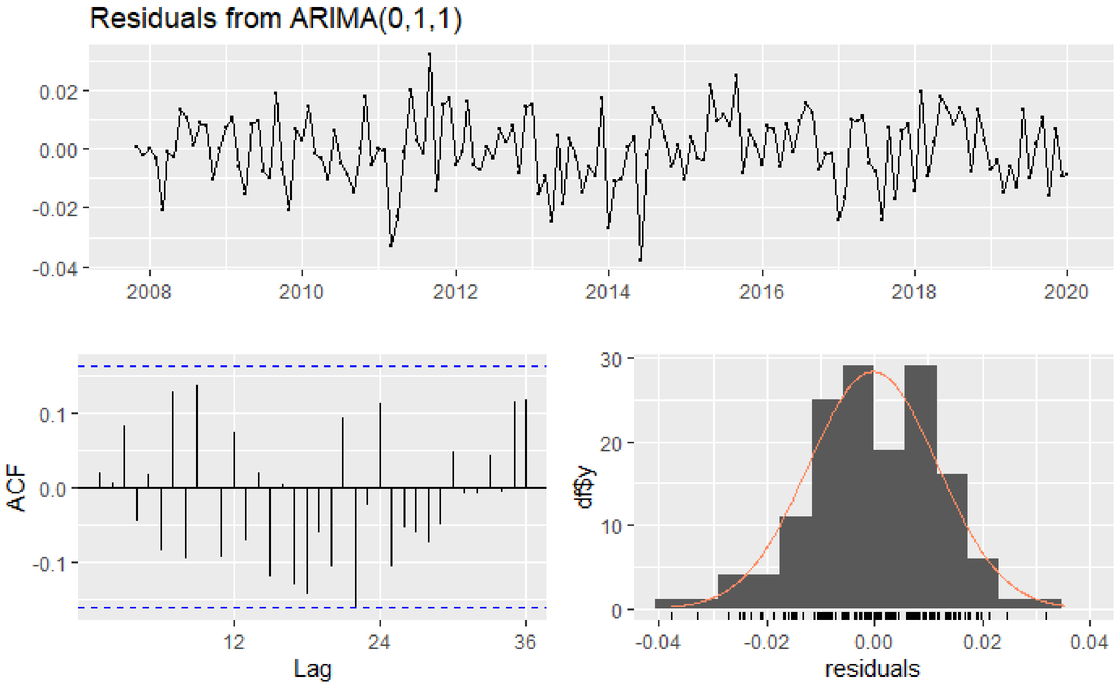

Figure S15: Residuals time series plot, residuals auto-correlation function (ACF) plot, and residuals histogram for adjusted ARIMAX(0,1,1) model for the outcome of current smoking among those aged 25+ years.

- Ljung-Box test results for residuals from adjusted ARIMAX(0,1,1) with non-zero mean: Q*=37.73, df=23, p=0.086 → no significant autocorrelation.

Table S27: Results for sensitivity analysis for current smoking among those aged 25 years and older as the outcome variable with model ARIMAX(0,1,1) and covariates the same as in the main analysis

Variables	Unadjusted model		Adjusted model	
	B (95% CI)	p-value	B (95% CI)	p-value
MA1	-0.864 (-0.936, -0.792)	<0.001	-1.000 (-1.040, -0.960)	<0.001
Standardised packaging (intervention effect, modelled using a step change)	-0.008 (-0.022, 0.006)	0.267	-0.002 (-0.012, 0.008)	0.718
Tobacco control policies (comprised score as dummy variable)	NA	NA	-0.001 (-0.015, 0.013)	0.886
Tax increases (transfer function, lag 0)	NA	NA	-0.140 (-0.221, -0.059)	0.001
Mass media expenditure (transfer function, lag 0)	NA	NA	-0.010 (-0.016, -0.003)	0.002
Use of prescription medication in quit attempt (transfer function, lag 0)	NA	NA	-0.001 (-0.135, 0.132)	0.987
Use of over-the-counter medication or behavioural support in quit attempt (transfer function, lag 0)	NA	NA	0.795 (0.452, 1.139)	<0.001
E-cigarette use (transfer function, lag 0)	NA	NA	0.166 (-0.083, 0.414)	0.191

References

1 Hammond D. Health warning messages on tobacco products: a review. *Tobacco Control* 2011;20(5):327-37. doi: 10.1136/tc.2010.037630

2 Kuipers MAG, Beard E, Hitchman SC, et al. Impact on smoking of England's 2012 partial tobacco point of sale display ban: a repeated cross-sectional national study. *Tobacco Control* 2017;26(2):141-48. doi: 10.1136/tobaccocontrol-2015-052724

3 Robertson L, McGee R, Marsh L, Hoek J. A systematic review on the impact of point-of-sale tobacco promotion on smoking. *Nicotine & Tobacco Research* 2014;17(1):2-17. doi: 10.1093/ntr/ntu168

4 Beard E, Brown J, Shahab L. Smoking prevalence following the announcement of tobacco tax increases in England between 2007 and 2019: an interrupted time–series analysis. *Addiction* 2022;117(9):2481-92. doi: 10.1111/add.15898

5 Beard E, Jackson SE, West R, et al. Population-level predictors of changes in success rates of smoking quit attempts in England: a time series analysis. *Addiction* 2020;115(2):315-25. doi: 10.1111/add.14837

6 Langley T, Szatkowski L, Lewis S, et al. The freeze on mass media campaigns in England: a natural experiment of the impact of tobacco control campaigns on quitting behaviour. *Addiction* 2014;109(6):995-1002. doi: 10.1111/add.12448

7 Kuipers MAG, Partos T, McNeill A, et al. Smokers' strategies across social grades to minimise the cost of smoking in a period with annual tax increases: evidence from a national survey in England. *BMJ Open* 2019;9(6):e026320. doi: 10.1136/bmjopen-2018-026320

8 Hyndman RJ, Khandakar Y. Automatic time series forecasting: the forecast package for R. *Journal of Statistical Software* 2008;27(3):1 - 22. doi: 10.18637/jss.v027.i03

9 Beard E, Marsden J, Brown J, et al. Understanding and using time series analyses in addiction research. *Addiction* 2019;114(10):1866-84. doi: 10.1111/add.14643

10 Metes DV. Visual, unit root and stationarity tests and their power and accuracy. Edmonton, Canada: Department of Mathematical and Statistical Sciences, University of Alberta, 2005.

11 Dickey DA, Fuller WA. Distribution of the estimators for autoregressive time series with a unit root. *Journal of the American statistical association* 1979;74(366a):427-31. doi: 10.1080/01621459.1979.10482531

12 Wang X, Smith K, Hyndman R. Characteristic-based clustering for time series data. *Data Mining and Knowledge Discovery* 2006;13(3):335-64. doi: 10.1007/s10618-005-0039-x

13 Wickham H, Averick M, Bryan J, et al. Welcome to the tidyverse. *Journal of Open Source Software* 2019;4(43):1686. doi: 10.21105/joss.01686

14 Survey: analysis of complex survey samples [program]. R package version 4.0 version. CRAN R-project, 2020.

15 tibbletime: time aware tibbles [program]. R package version 0.1.6 version. CRAN R-Project, 2020.

16 Hyndman RJ, Athanasopoulos G, Bergmeir C, et al. forecast: forecasting functions for time series and linear models, 2023.

17 Wickham H. ggplot2: elegant graphics for data analysis. New York, United States: Springer-Verlag 2016.

18 tseries: time series analysis and computational finance [program]. R package version 0.10-54 version. CRAN R-project, 2023.

19 Chan K-S, Ripley B. Time Series Analysis - package ‘TSA’. CRAN repository, 2022.

20 Collis D. Social grade: A classification tool–Bite sized through piece. Ipsos MediaCT. London, United Kingdom, 2009:1-6.
